# Immunological dimensions of neuroinflammation and microglial activation: exploring innovative immunomodulatory approaches to mitigate neuroinflammatory progression

**DOI:** 10.3389/fimmu.2023.1305933

**Published:** 2024-01-08

**Authors:** Lucas Fornari Laurindo, Jefferson Aparecido Dias, Adriano Cressoni Araújo, Karina Torres Pomini, Cristiano Machado Galhardi, Claudia Rucco Penteado Detregiachi, Luíza Santos de Argollo Haber, Domingos Donizeti Roque, Marcelo Dib Bechara, Marcela Vialogo Marques de Castro, Eliana de Souza Bastos Mazuqueli Pereira, Ricardo José Tofano, Iris Jasmin Santos German Borgo, Sandra Maria Barbalho

**Affiliations:** ^1^ Department of Biochemistry and Pharmacology, School of Medicine, Faculdade de Medicina de Marília (FAMEMA), Marília, São Paulo, Brazil; ^2^ Department of Biochemistry and Pharmacology, School of Medicine, Universidade de Marília (UNIMAR), Marília, São Paulo, Brazil; ^3^ Postgraduate Program in Structural and Functional Interactions in Rehabilitation, School of Medicine, Universidade de Marília (UNIMAR), Marília, São Paulo, Brazil; ^4^ Department of Anatomy, School of Medicine, Universidade de Marília (UNIMAR), Marília, São Paulo, Brazil; ^5^ Department of Biological Sciences (Anatomy), School of Dentistry of Bauru, Universidade de São Paulo (FOB-USP), Bauru, São Paulo, Brazil; ^6^ Department of Biochemistry and Nutrition, School of Food and Technology of Marília (FATEC), Marília, São Paulo, Brazil

**Keywords:** neuroimmunology, neuroinflammation, neuroinflammatory disorders, neurodegenerative diseases, microglia, microglial activation, immune system, Alzheimer’s disease

## Abstract

The increasing life expectancy has led to a higher incidence of age-related neurodegenerative conditions. Within this framework, neuroinflammation emerges as a significant contributing factor. It involves the activation of microglia and astrocytes, leading to the release of pro-inflammatory cytokines and chemokines and the infiltration of peripheral leukocytes into the central nervous system (CNS). These instances result in neuronal damage and neurodegeneration through activated nucleotide-binding domain and leucine-rich repeat containing (NLR) family pyrin domain containing protein 3 (NLRP3) and nuclear factor kappa B (NF-kB) pathways and decreased nuclear factor erythroid 2-related factor 2 (Nrf2) activity. Due to limited effectiveness regarding the inhibition of neuroinflammatory targets using conventional drugs, there is challenging growth in the search for innovative therapies for alleviating neuroinflammation in CNS diseases or even before their onset. Our results indicate that interventions focusing on Interleukin-Driven Immunomodulation, Chemokine (CXC) Receptor Signaling and Expression, Cold Exposure, and Fibrin-Targeted strategies significantly promise to mitigate neuroinflammatory processes. These approaches demonstrate potential anti-neuroinflammatory effects, addressing conditions such as Multiple Sclerosis, Experimental autoimmune encephalomyelitis, Parkinson’s Disease, and Alzheimer’s Disease. While the findings are promising, immunomodulatory therapies often face limitations due to Immune-Related Adverse Events. Therefore, the conduction of randomized clinical trials in this matter is mandatory, and will pave the way for a promising future in the development of new medicines with specific therapeutic targets.

## 1 Introduction

The steady rise in life expectancy has increased the prevalence of age-related neurodegenerative diseases. Significantly, the sustenance of neuronal functions and the preservation of cognitive capabilities hinge on the accessibility of elevated energy levels to meet the neurons’ demands (1, 2). As a result, the substantial oxygen consumption by neurons may signify the heightened susceptibility of the brain to oxidative stress from reactive oxygen species (ROS) and ensuing inflammation (3, 4).

Neuroinflammation is characterized by an inflammatory reaction within the brain or spinal cord (5). It is orchestrated by releasing cytokines, chemokines, reactive oxygen species, and secondary messengers (6). These signaling molecules are generated by various cell types, including resident central nervous system (CNS) glia (microglia and astrocytes), endothelial cells, and immune cells originating from the periphery (7). The consequences of these neuroinflammatory responses encompass immune, physiological, biochemical, and psychological implications (8). At first, in its temporary phase, neuroinflammation functions as a safeguarding mechanism. Nevertheless, findings from both clinical and pre-clinical investigations indicate that prolonged or maladaptive neuroinflammation assumes a central role as a pathological catalyst in various neurological conditions. These encompass neurodegenerative diseases, psychiatric disorders, pain syndromes, stroke, and traumatic brain injury (9–11).

Mitigating neuroinflammation to diminish disease severity and enhance individual outcomes is considered a strategy against neurodegeneration. From a molecular standpoint, several conventional, targetable elements are involved in neuroinflammation, including enzymes, receptors, and ion channels (12, 13). While elevated expenses and restricted efficacy potentially raise concerns about using conventional drugs to inhibit neuroinflammation targets, there is still a substantial opportunity to investigate novel therapies for alleviating neuroinflammation in CNS diseases, possibly even before their onset (14). In this scenario, the interest in innovative anti-inflammatory and neuroprotective immunomodulatory therapies increased.

No current reviews emphasized the potential of cutting-edge and innovative immunomodulatory therapies as controllers of neuroinflammation, covering models of microglial activation, neurodegeneration, and brain aging. The present study also stands out for its comprehensive examination and detailed exploration of the fundamental inflammatory signaling pathways central to these processes. Trewin et al. (15) exclusively concentrated on the impacts of immunotherapy on encephalitis disability outcomes, overlooking the resolution or decrease of neuroinflammation through the application of immunotherapeutic approaches. A similar situation can be exemplified in the field of neuro-oncology by Majd et al. (16) and neurodegeneration by Wang & Colonna (17), which exclusively addressed Alzheimer’s. Xu et al. (18) delved into microglia’s functional and phenotypic diversity and its relevance to microglia-based therapies for Alzheimer’s Disease. Nevertheless, their emphasis was profoundly on the phagocytic microglial phenotype derived from Alzheimer’s Disease, neglecting a profound discussion of the other inflammatory signaling pathways linked to microglial activation in the context of the disease and other neuroinflammatory diseases. Likewise, Araújo et al. (19) explored the intricate mechanisms of the microglial phenotype, specifically in Parkinson’s Disease. However, these authors seem to concentrate solely on the peripheral response within the Parkinson’s neuroenvironment and did not extensively delve into the inflammatory signaling pathways implicated. Finally, Brambilla (20) concentrated on the astrocyte response in both multiple sclerosis and experimental autoimmune encephalomyelitis. While addressing a single disease, the author extensively explored the intricate mechanisms of astrocytes’ reaction to soluble mediators (such as cytokines and chemokines), the regulation of oxidative stress, and the preservation of blood-brain barrier (BBB) integrity and function. Nevertheless, there was not an in-depth discussion of the inflammatory pathways involved. The present study seeks to compile a comprehensive review encompassing these facets to bridge this gap in knowledge from lab to clinic.

## 2 Materials and methods

### 2.1 Focal question

The primary questions driving this review are: “*What are the Neuroimmunological Aspects of Neuroinflammation and Microglial Activation in Alzheimer’s Disease, Parkinson’s Disease, and Multiple Sclerosis?’’ and ‘‘What are the Current Trends in Mitigating Neuroinflammation with Innovative Immunomodulatory Options?”*.

### 2.2 Inclusion and exclusion criteria

This comprehensive review systematically investigated both *in vitro* and *in vivo* studies employing innovative immunomodulatory therapies for intervening in neuroinflammation. Carefully selected criteria were applied to ensure the inclusion of studies directly relevant to the research question, with a focus on full-text articles. Conversely, exclusion criteria were employed to filter out reviews, poster presentations, case reports, and editorials, eliminating studies that did not align with the review’s objectives or exhibited a high risk of bias.

To categorize and synthesize the studies, a systematic approach was implemented. All identified studies underwent title screening using automated tools, with a subsequent critical examination of their abstracts. At this stage, duplicates were also removed. Subsequently, studies meeting the initial criteria underwent a more thorough assessment involving comprehensive reading and interpretation. This process facilitated a detailed evaluation of study quality, relevance, and applicability. Following this comprehensive assessment, the studies underwent bias assessment to ensure the review process’s robustness further.

### 2.3 Language

This review exclusively included studies conducted in English.

### 2.4 Databases

We conducted a comprehensive search across reputable databases, including PubMed, EMBASE, and COCHRANE, with the last access on 29 November 2023. The employed Mesh terms were thoughtfully selected to optimize the search process, encompassing terms such as “Neuroinflammation,” “Neuroinflammation Intervention,” “Alzheimer’s Disease,” “Parkinson’s Disease,” and “Multiple Sclerosis.” These terms were combined with “Microglia,” “Microglia Activation,” “Immunomodulation,” and “Immunomodulatory Approaches” using the Boolean operator ‘and.’ The deliberate choice of these Mesh terms aimed at identifying relevant *in vitro* and *in vivo* studies aligning with the review’s objectives. The absence of predefined filters or restrictions during the identification of included studies was intentional, ensuring a comprehensive and unbiased search strategy. This approach allowed for an exhaustive exploration of the available literature. The determination of whether a study met the inclusion criteria followed a systematic and transparent approach. Two independent reviewers, *L.F. Laurindo* and *S.M. Barbalho*, actively participated in the screening process. In instances of disparities in their initial assessments, a third reviewer, *K.T. Pomini*, was engaged to facilitate resolution.

### 2.5 Study selection

Due to the current absence of clinical studies exploring the impacts of innovative immunomodulatory approaches on individuals with neuroinflammatory diseases, our review deliberately narrowed its focus. Instead, we dedicated our attention to an exhaustive analysis of *in vitro* and *in vivo* studies. This approach gave us insights into the mechanisms, efficacy, and potential therapeutic applications of these last-researched immunomodulatory interventions in neuroinflammation. We aimed to offer a comprehensive overview of the existing knowledge base by concentrating on preclinical studies. These in-depth examinations of cellular and animal models provide valuable insights into the potential translational relevance of these innovative approaches for human neuroinflammatory conditions. While acknowledging the preliminary nature of such studies, this review lays the groundwork for future clinical investigations by synthesizing and critically evaluating the available preclinical evidence.

### 2.6 Data extraction

The deliberately expansive search strategy employed for both *in vitro* and *in vivo* studies was designed without temporal restrictions. This intentional approach was chosen to embrace diverse, relevant research across different timeframes and to include immunomodulatory interventions that, primarily due to cost and other ethical constraints, have not yet been translated into clinical research. By refraining from imposing specific temporal restrictions, our objective was to encompass the entirety of the available literature, facilitating a thorough exploration of the subject matter. This inclusive strategy proved crucial in identifying potential trends, historical perspectives, and evolving methodologies pertinent to the research question. Throughout its construction, this review strictly adhered to the guidelines outlined in the Preferred Reporting Items for Systematic Reviews and Meta-Analyses (PRISMA) (21). This adherence ensures transparency and rigor in the review process, aligning with established standards for systematic reviews and enhancing the reliability of our findings.

### 2.7 Quality assessment

Two proficient evaluators (*R.J. Tofano* and *M.D. Bechara)* who underwent training in the Joanna Briggs Institute (JBI) Checklist Critical Appraisal Tool for Systematic Reviews’ Quality (22, 23) were entrusted with the responsibility of conducting the quality assessment. The JBI, an esteemed global research institution within the Faculty of Health and Medical Sciences at the University of Adelaide in South Australia, has devised a set of 11 standardized questions aimed at streamlining the evaluation process for systematic reviews or meta-analyzes. These reviewers were guided to provide responses categorized as “Yes,” “No,” or “Unclear,” with the option “Not Applicable” (NA) available in specific instances. Comprehensive definitions and illustrative examples were developed to facilitate practical scale use, offer guidance, and enhance clarity for the reviewers. These guidelines are meticulously crafted to ensure a consistent and accurate assessment of each item on the scale. In instances where discrepancies arose between the initial two reviewers, a third independent reviewer (*C.R.P. Detregiachi*) was engaged to mediate and facilitate resolution, further enhancing the reliability and objectivity of the quality assessment process.

## 3 Nuclear Factor Kappa B (NF-κB): a pioneer shaping the landscape of neuroinflammation and neurodegenerative conditions

Nuclear Factor Kappa B (NF-κB), an inducible transcription factor also known as the nuclear factor of the κ-chain in B-cells, plays a crucial role in regulating a wide array of genes involved in both developmental and immune-modulatory processes, not limited to inflammation (24). NF-κB comprises five members, namely ReIB, c-ReI, ReIA (p65), NF-κB1 (p50), and NF-κB2 (p52), all sharing similar structures (25). These components facilitate the transcription process of various inflammatory agents by binding to specific deoxyribonucleic acid (DNA) elements known as kB enhancers, forming different hetero- and homo-dimers (26). In activated cells, NF-κB-related proteins are sequestered in the cytoplasm by a group of inhibitory proteins known as IkB, characterized by ankyrin repeats. IkBα has been extensively studied (27–30). At the molecular level, numerous proinflammatory cytokines and factors like interleukin (IL)-33, lymphotoxin-β, IL-1β, IL-12, granulocyte macrophage-colony stimulating factor (GM-CSF), tumor necrosis factor (TNF)-α, and IL-17 initiate NF-κB signaling. Additionally, microbial antigens such as CpG-DNA, enterotoxins, flagellin, and lipopolysaccharide (LPS), as well as viral agents and proteins, and various receptor ligands like CD40L, Fas ligand (FasL), TNF-related apoptosis-inducing ligand (TRAIL), bone morphogenetic proteins (BMP)-2, B-cell activating factor (BAFF), hepatocyte growth factor (HGF), and BMP-4, can also activate NF-kB (31–34). Furthermore, components released during cell rupture, including extracellular ribonucleic acid (RNA) molecules, high mobility group box 1 (HMGB1), extracellular DNA molecules, and damage-associated molecular patterns (DAMPs), as well as several eukaryotic parasites like *Leishmania* and *Candida albicans*, can trigger NF-κB activation (35–39). Finally, physiological stressors like hyperglycemia, endoplasmic reticulum (ER) stress, oxidative stress (OS), and acidic pH, along with mechanical stressors resulting from the presence of altered proteins and particles, such as oxidized low-density lipoprotein cholesterol (LDL), amyloid protein fragments, and advanced glycation end products (AGEs), as well as ionizing radiation and ultraviolet (UV) light exposure, also activate NF-κB (25, 40, 41).

NF-κB activation typically involves two main pathways: the canonical and non-canonical (or alternative) (25). In the canonical pathway, NF-κB is primarily triggered by ligands binding to receptors of proinflammatory cytokines such as the T cell receptor (TCR), members of the TNF receptor superfamily (TNFRSF), pattern-recognition receptors (PRRs), and the B cell receptor (BCR) (42–44). This conventional pathway initiates with the controlled and inducible degradation of IkBα, facilitated by precise phosphorylation at specific sites carried out by a multi-subunit IkB kinase (IKK) complex (45). Various molecules and stressors, including proinflammatory cytokines, growth factors, microbial components, and mitogens, can activate the IKK complex (46). Molecularly, the IKK complex consists of two catalytic subunits, IKKα and IKKβ, along with a regulatory subunit known as NF-κB essential modulator (NEMO) or IKKγ. Once activated, the IKK complex phosphorylates IKKα at two serine residues in the N-terminal region. These residues lead to the ubiquitin-dependent degradation of IkKα in the proteasome, resulting in the rapid and transient translocation of canonical NF-κB members from the cytoplasm to the nucleus. The canonical NF-κB dimers primarily comprise p50/ReIA and p50/c-ReI (25, 39, 47, 48).

The canonical activation of NF-κB is central in orchestrating and advancing immunological responses. It is instrumental in triggering various facets of the immune system (49). In the context of inflammation, NF-κB fosters the heightened production of proinflammatory cytokines, chemokines, and adhesion molecules, while also regulating immune cells’ differentiation, proliferation, morphogenesis, and apoptosis (50, 51). Specifically, dendritic cells are prompted to mature, enabling them to present antigens and initiate immune responses [29] efficiently. Within the influence of NF-κB signaling, T cells also undergo differentiation and activation driven by inflammation (52). This signaling pathway is pivotal in forming memory T cells, which play a critical role in long-term immunity. Factors such as RAR-related orphan receptor gamma transcription (RORγt), IL-23, and IL-12 are intimately linked within this facet of the NF-κB signaling (53–55). Moreover, NF-κB activation spurs macrophages to produce substantial quantities of proinflammatory cytokines and chemokines and prompts them to polarize into the M1 phenotype, known for its proinflammatory functions (56–59). Neutrophils are also influenced by NF-κB signaling, resulting in their anti-apoptotic state and extensive recruitment to sites of inflammation, thereby bolstering the immune response (60–62).

On the other hand, the non-canonical (alternative) activation of NF-κB is more specialized and reacts to a distinct set of stimuli. This pathway is set in motion by ligands binding to a subset of TNFRSF (63). In contrast to the canonical path, the non-canonical route involves processing the precursor protein NF-κB2 (p100) (64). This processing of p100 entails the degradation of its C-terminal IkB-like structure, leading to the generation of a mature form of NF-κB2 known as p52 (65). Subsequently, this mature NF-κB2 p52 relocates into the nucleus in conjunction with ReIB. A crucial player in orchestrating this degradation process is a signaling molecule called NF-κB-inducing kinase (NIK) (66, 67). NIK effectively instigates the activation of IKKα and works in collaboration with it to facilitate the phosphorylation of p100 (34, 68). Previous research has established that non-canonical NF-κB activation functions as an additional signaling axis that integrates precise adjustments and enhances immune responses alongside the conventional pathway in overseeing specific adaptive roles within the acquired immunity (25, 39, 69, 70).

From a biological standpoint, NF-κB is pivotal in coordinating cellular reactions to various stressors and challenging environments (71). As mentioned, NF-κB prompts diverse cells to adapt to threats and initiate their defense mechanisms upon activation and signaling. This enables them to counteract the danger effectively and prevent cellular death (72). The ultimate objective is to restore the body’s original physiological state and uphold cellular equilibrium, known as homeostasis. To accomplish this, NF-κB targets a broad spectrum of genes, either enhancing their expression or inducing them to evoke protective responses (73). However, despite its critical function in safeguarding the body, NF-κB’s proinflammatory nature also implicates it in the pathophysiology and progression of various inflammatory conditions, including neuroinflammatory disorders (74).

To significantly instigate inflammatory diseases, NF-κB operates by activating and regulating various inflammasomes (75). Inflammasomes are composed of multi-protein complexes within cells that assemble and activate caspases not only in response to pathogen-associated molecular patterns (PAMPs) but also to DAMPs (76). Inflammasomes are well-recognized pivotal components of the innate immune system. They typically include a ligand-sensing receptor, often belonging to the nucleotide-binding domain leucine-rich repeat (NLR) family (such as NLRP1, NLRP3, NLRC4, or AIM2), an adaptor protein (usually the apoptosis-associated speck-like protein containing a CARD domain [ASC]), and a pro-caspase (typically pro-caspase 1) (77). Upon receiving the appropriate stimulus, these receptors form oligomers and enlist pro-caspase 1 with the assistance of the ASC protein. (78). Subsequently, pro-caspase 1 transforms into active caspase 1, which cleavages pro-IL-1β and pro-IL-18, producing their mature forms (79). Indeed, the controlled release of IL-18 and IL-1β can initiate numerous inflammatory processes stemming from the inflammasome (80, 81). Presently, NLRP3 stands out as the most extensively studied inflammasome. It is composed of NLRP3, ASC, the essential regulatory protein never in mitosis gene A (NIMA)-related kinase 7 (NEK7), and pro-caspases 1 (82, 83). The dysregulation and disruption of NLRP3 can lead to subsequent NF-κB hyperactivation, giving rise to various inflammatory diseases (84–86).

In the face of neuronal challenges, NF-κB exhibits a sustained level of activity in the cell bodies of neurons, offering protection against various injuries and regulating neuroinflammatory responses (87). Furthermore, NF-κB transcription factors are abundant in numerous cells of the CNS, including glial and endothelial cells of cerebral blood vessels, where they play a regulatory role in the neuronal environment and perform diverse functions (88, 89). Additionally, NF-κB activation is associated with several CNS diseases (90). Interestingly, the multifarious processes of NF-κB in the CNS are contingent on the specific subunits involved in NF-κB dimer formation within brain cells. Notably, the expression of RelA and c-Rel has distinct effects on neuronal survival, with the presentation of c-Rel being particularly crucial in mitigating apoptosis and age-related behaviors within the CNS (91).

NF-κB transcription factors exhibit a constitutive expression in the brain, with baseline levels higher than those found in peripheral tissues (92). Studies indicate that NF-κB shows consistent activation in CNS glutamatergic neurons, including specific regions like the hippocampus (comprising pyramidal neurons and granule cells of CA1 and CA3) and the cerebral cortex (specifically layers 2, 4, and 5) (93). At the molecular level, this ongoing NF-κB activity in glutamatergic neurons of the cerebral cortex and hippocampus can be inhibited by N-methyl-D-aspartate and AMPA glutamate receptor antagonists, underscoring its regulation by basal synaptic transmission (88, 94). This constitutive NF-κB activity has also been noted in various brain regions of rodents, including the amygdala, cerebellum, hypothalamus, hippocampus, cerebral cortex, and olfactory lobes (88).

Moreover, inducible NF-κB can also be detected within synapses, and the activation of the glutamatergic system induces the retrograde movement of the p65 protein from synapses to the nucleus (95, 96). This implies that NF-κB plays a role in converting brief synaptic signals into enduring changes in gene expression (97). Additionally, the activated IKK and its product, phosphorylated IκBα, have been identified at the axon initial segment—the site where action potentials originate—suggesting that constitutive NF-κB activation is involved in neuronal information processing (98–100).

In summary, in the CNS, NF-κB assumes pivotal functions with various biological processes, including neurogenesis, neuritogenesis, and synaptic plasticity, all intricately connected to learning and memory (88). Additionally, multiple studies have furnished evidence that NF-κB activation can confer a degree of neuronal safeguarding against diverse forms of harm, such as excitotoxicity, oxidative stress, and the harmful effects of excessive Aβ peptide, effectively acting as a cellular defense mechanism (101–103).

## 4 NLR (nucleotide-binding domain and leucine-rich repeat containing) family pyrin domain containing 3 (NLRP3) inflammasome on neuroinflammation: investigating a potential therapeutic avenue for neuroinflammatory conditions

Inflammasomes are complex protein assemblies found within the cytoplasm, serving a dual role in activating IL-1β and IL-18 and triggering pyroptosis (104). Their principal function is to initiate and sustain the innate immune response against various stressors, whether from within the body or externally (105). Structurally, NLRs comprise distinctive domains, including an N-terminal effector domain, a C-terminal leucine-rich repeat (LRR) region, and a central nucleotide-binding domain (NBD/NOD/NACHT). While NACHT and LRR are conserved across all NLRs except for NLRP10, the N-terminal effector domain exhibits variability (106). This variability allows NLRs to engage with different partners and recruit various integrators. Beyond their well-known role in activating the NF-kB inflammatory pathway, NLRs also play a part in mitogen-activated protein kinase (MAPK) signaling, antigen presentation, cytosolic signal transduction complexes assembly, and embryonic development (107, 108).

Within the NLR family of inflammasomes, NLRP3 emerges as a distinctive receptor (109). Unlike its counterparts, NLRP3 undergoes indirect activation triggered by pathogenic and sterile proinflammatory cues. When exposed to bacterial and viral PAMPs such as nucleic acids, LPS, nigericin, and gramicidin, along with DAMPs, ROS, extracellular adenosine triphosphate (ATP), potassium efflux, and metabolic crystals, the NLRP3 domain is prompted into action (110).

At the molecular level, the activation of NLRP3 relies on three key components: a sensor (NLRP3), an adaptor (ASC or PYARD), and an effector (caspase-1). This process encompasses two crucial phases: priming and activation (111). During the priming phase, there is an essential upregulation of NLRP3, pro-IL-1β, and caspase-1. This upregulation is initiated by activating PRRs and cytokine receptors, including toll-like receptors (TLRs) and IL-1 receptors (IL-1R), in response to PAMPs and DAMPs, respectively. Subsequently, the activation phase of NLRP3 is prompted by either endogenous molecules, such as DAMPs, or external stimuli, like PAMPs, K+, and Cl− ions efflux, or the influx of Ca2+ (77).

Upon detecting hazardous signals, typically recognized via the LRR domain, individual NLRP3 monomers initiate the oligomerization process. In this phase, they engage with pyrin domains, specifically the apoptosis-associated speck-like proteins containing a CARD (PYCARD). This interaction occurs through homophilic connections, leading to the recruitment of cysteine protease pro-caspase-1 by ASC. ASC, acting as an adaptor, effectively utilizes its caspase recruitment domain (CARD) to facilitate this recruitment. As a result, caspase-1 undergoes autocatalysis and subsequent activation, producing proinflammatory cytokines IL-1β and IL-18 and instigating pyroptosis, a form of programmed cell death (107, 112).

Indeed, NLRP3 has been observed to engage with nucleotide-binding oligomerization domain 2 (NOD2) through a CARD-dependent mechanism (113). This interaction mediates explicitly the processing of the prominent NLRP3-associated proinflammatory cytokine, IL-1β (114). Unlike other cytokines within the IL-1 family that can attenuate inflammation, IL-1β actually promotes it. This cytokine plays a pivotal role in coordinating proinflammatory responses across various tissues, thereby contributing to the pathogenesis of numerous systemic inflammatory disorders. Furthermore, IL-1β is closely associated with leukocytosis and heightened levels of acute phase inflammatory proteins (115).

Verily, the activation of the NLRP3 inflammasome is intricately connected to the development of a range of inflammatory and immunomodulated conditions such as diabetes, inflammatory bowel diseases, and atherosclerosis (77). Nevertheless, an excessive activation level is necessitated for NLRP3 to precipitate illness (116). Given its multifaceted functions, it is imperative to carefully modulate NLRP3 to avert undesirable disease pathways and to safeguard the organism from the harm caused by excessive inflammation.

The NLRP3/caspase-1/IL-1 axis has emerged as a pivotal signaling pathway within the innate immune system of the CNS (117). Caspase-1 is notably abundant in disorders associated with neuroinflammation (118). Furthermore, IL-1β and IL-18 have been implicated in the onset of neuroinflammation (119, 120). Research has demonstrated heightened levels of IL-1β and IL-18 in the cerebrospinal fluid, brain tissue, and plasma of individuals affected by CNS infections, brain injuries, Alzheimer’s disease (AD), and multiple sclerosis (MS) (110). Following binding to their respective receptors on microglial cells, astrocytes, neurons, and endothelial cells, IL-1β and IL-18 set off a complex cascade of signaling events, ultimately leading to the subsequent expression of various inflammation-related genes (121). Importantly, these cytokine-mediated processes have been associated with cognitive decline and enduring neuropsychiatric conditions (122–124).

Furthermore, IL-1β signaling plays a crucial role in initiating and sustaining inflammatory responses within the CNS in reaction to various detrimental stimuli (125). This cytokine also impacts the integrity of the BBB, directly infiltrating peripheral immune cells into the CNS (126, 127). Additionally, while stimulating the activation of microglia and astrocytes, IL-1β also activates T cells that have infiltrated the CNS, fostering the production of proinflammatory factors like IL-6 and TNF-α, as well as neurotoxic mediators (128). Moreover, IL-1β indirectly attracts leukocytes by heightening the expression of chemokines, and experimental studies have indicated that IL-1β overexpression contributes to neuronal injury by modulating glutamate excitotoxicity (110, 129).

Conversely, IL-18 predominantly provokes T-helper (Th) cell-mediated immune responses, instigating the generation of adhesion molecules, proinflammatory cytokines, and chemokines in natural killer, Th1, and B cells. Additionally, IL-18 initiates signaling pathways in microglia, resulting in elevated expression of caspase-1, matrix metalloproteinases, and production of proinflammatory cytokines. Furthermore, IL-18 amplifies FasL expression in glial cells, exacerbating Fas-mediated neuronal cell death in situations of neuroinflammation (110, 130–132).

Evidently, pyroptosis, a highly inflammatory and programmed cell death intricately linked to NLRP3 activation, is exclusively orchestrated by activated caspase-1, distinguishing it from necrosis and apoptosis (133). Recently, pyroptosis has been observed in both glial cells and neurons (134). This type of cellular demise results in a swift rupture of the plasma membrane, leading to an abundant release of proinflammatory cytokines and chemokines such as TNF-α, IL-1β, IL-6, and CX3C-chemokine ligand 3. This exacerbates the inflammatory mediator-induced neuronal death (135, 136). It is worth noting that these elements draw immune cells from the bloodstream to the sites of inflammation, intensifying the inflammatory responses and resulting in significant tissue damage in the CNS, especially under neuropathological conditions (137, 138).

## 5 Harnessing the power of nuclear factor erythroid 2-related factor 2 (Nrf2) for combating oxidative stress and neuroinflammation in neurodegenerative disorders

Nrf2 is a member of the vertebrate Cap’n’Collar (CNC) transcription factor subfamily, a subset of the basic leucine zipper (bZip) transcription factors (139, 140). This subfamily encompasses additional members such as nuclear factor E2-related factors 1 and 3 (Nrf1 and Nrf3) and p45 NF-E2 (141).

At the molecular level, Nrf2 is crucial in overseeing the expression of many mammalian antioxidant genes under normal conditions and when triggered. Its activation is prompted by diverse stressors, including mild oxidative or electrophilic stress (142). Notably, several chemical agents are recognized for their ability to enhance internal antioxidants by activating Nrf2. Indeed, the remarkable feature of Nrf2 lies in its responsiveness, allowing it to dynamically react to environmental stressors (141).

In theory, many cellular elements govern the stability of the Nrf2 protein, consequently influencing its movement into the nucleus. Among these factors, Keap1 emerges as the foremost player (140, 143). Typically, Nrf2 is situated in the cytosol, forming a complex with Keap1 (Kelch-like ECH-associated protein 1), also recognized as an inhibitor of Nrf2 (INrf2), which is an actin-binding protein (144). Keap1 is pivotal in modulating Nrf2 function and exists in cellular dimers. Functioning as a substrate linker, Keap1 facilitates the binding of the Cul3/Rbx1-based E3-ubiquitin ligase complex to Nrf2, leading to the continuous ubiquitination of Nrf2 and subsequent degradation through the proteasome pathway (145, 146). This continual degradation of Nrf2 under normal conditions maintains low levels of Nrf2, thereby restricting the expression of Nrf2-regulated antioxidants (147). However, in the presence of mild oxidative or electrophilic stress or exposure to chemical inducers, Nrf2 dissociates from Keap1. This event leads to its stabilization and subsequent translocation into the cell nuclei (148). Once in the nuclei, Nrf2 interacts with various protein factors, including small Maf (sMaf), and binds to antioxidant response elements (ARE), consequently facilitating heightened transcription of antioxidant genes (149). In mammals, including humans, the Keap1-Cul3-Rbx1 axis is the foremost regulatory mechanism governing Nrf2 activity (141).

Several mechanisms have been proposed to clarify the separation of Nrf2 from Keap1 during periods of stress (150). Of particular note, two mechanisms have received substantial attention: the oxidation of cysteine residues in Keap1 and the binding of p62 to Keap1 (140, 151, 152). Keap1 is a protein-rich in cysteine, with specific cysteine residues as sensors responsive to electrophiles and ROS (153). Alterations to particular cysteine sulfhydryl groups, particularly Cys151, Cys273, and Cys288, within the Keap1 protein trigger the dissociation of Nrf2 from Keap1. As a result, the Keap1-Nrf2 system is acknowledged as a pivotal thiol-based sensor-effector mechanism crucial for maintaining cellular redox balance (141).

Also known as sequestosome 1 (SQSTM1), p62 is a protein that binds to ubiquitin and guides protein aggregates toward degradation via the autophagic pathway (154, 155). It competes with Nrf2 for Keap1 binding, which leads to Keap1 degradation and stabilizes Nrf2. Notably, the promoter of the p62 gene contains an ARE, making it a target gene for Nrf2. This creates a positive feedback loop where Nrf2 induces ARE-driven gene transcription, thereby increasing p62 levels (141, 156, 157). In simpler terms, p62 enhances Nrf2 protein stability, and in return, Nrf2 triggers the expression of the p62 gene, resulting in further elevations in p62 levels (140, 156, 158).

The Nrf2 gene promoter also incorporates a binding site for NF-κB, with NF-κB subunits p50 and p65 promoting the transactivation of the Nrf2 gene. This elucidates how Nrf2 can be triggered in response to inflammatory cytokines that activate NF-κb (141). Intriguingly, while NF-κB initiates Nrf2 activation, conversely, Nrf2 activation dampens NF-κB signaling, indicating a reciprocal interaction between these two pathways (159). Consequently, the inhibition of NF-κB signaling by Nrf2 may, at least in part, contribute to the anti-inflammatory function of Nrf2 activators like sulforaphane (160, 161).

The precise mechanism by which Nrf2 inhibits NF-κB signaling is not fully understood. Nonetheless, it is proposed that the activation of Nrf2 may alter the cellular redox state toward a more reduced condition facilitated by heightened antioxidant expression (141). This shift toward a more reducing environment subsequently diminishes NF-κB activation, as it is less prone to activation under such circumstances (162).

Interestingly, evidence suggests autoregulation of Nrf2 signaling through two distinct mechanisms (163). Firstly, due to ARE-like sequences in the Nrf2 gene promoter region, Nrf2 can activate its gene expression, leading to an increased production of the Nrf2 protein (164). This establishes a positive feedback loop, reinforcing Nrf2 signaling (141). Secondly, Nrf2 can also stimulate the expression of the Keap1 gene, subsequently facilitating its degradation (165). This creates a negative feedback loop, preventing excessive Nrf2 expression and maintaining controlled Nrf2 signaling (141).

To summarize, Nrf2 acts as a transcription factor, driving the expression of a range of genes crucial for cellular protection and detoxification (166). Its primary role is as an inherent defense mechanism against oxidative harm (167). Additionally, the Nrf2-ARE pathway governs an array of antioxidant enzymes and proteins tasked with detoxifying, repairing, removing damaged tissues, and alleviating inflammation (168).

In light of this, the Nrf2-ARE pathway has garnered extensive attention in neurodegenerative conditions, showcasing its protective roles against neuroinflammation (169). According to existing literature, aging is linked with significant global health challenges, particularly neurodegenerative disorders, as the body’s defenses against oxidative stress and inflammation weaken (170). As a result, the pharmacological activation of Nrf2 holds promise as a therapeutic strategy for tackling neurodegenerative disorders characterized by an overproduction of reactive oxygen species and inflammation (171, 172). Delving into the specifics, Nrf2’s mechanisms in countering neurodegeneration and neuroinflammation align with what was previously mentioned. This could potentially be attributed to its activation leading to the bolstering of antioxidant defenses, suppression of inflammation (including the transcriptional repression of proinflammatory cytokines like TNF-α, IL-1, IL6, IL-8, and monocyte chemoattractant protein-1 (MCP-1), from microglia, macrophages, monocytes, and astrocytes), improvement of mitochondrial function (which aids in safeguarding proper mitochondrial function from ROS generation and shielding cells from toxins released by mitochondria), and the maintenance of protein balance, ultimately mitigating several pathological processes implicated in neurological diseases (172–175).

In neurodegenerative disorders, the activity of Nrf2-HO-1 experiences a decline (171, 176). Recent findings highlight the neuroprotective effects associated with Nrf2-mediated induction of heme oxygenase-1 (HO-1) in diverse pathological conditions such as Alzheimer’s disease, Parkinson’s disease, and others (177–179). It is established that Nrf2 intricately regulates the pivotal enzyme, HO-1 (180). Given this context, encouraging strategies to address neurodegenerative disorders may involve activating Nrf2 and elevating HO-1 levels in microglia, as these approaches have demonstrated potential benefits in prior studies (175).

Various technical and methodological strategies aid investigations into Nrf2’s role in combating neuroinflammation. Specifically, Nrf2 hinders the transcription of genes responsible for proinflammatory cytokines in response to inflammation-inducing factors like LPS exposure. This characteristic lends itself well to studying Nrf2 in neuroinflammatory conditions, given that many neuroinflammation models involve LPS-induced microglial cells (175, 181, 182).

The interaction between the NF-κB and Nrf2-ARE systems, where Nrf2 activation potentially dampens NF-κB activity, has been suggested as a mechanism to counteract neurodegenerative and neuroinflammatory disorders (159, 183, 184). The idea is that by boosting the ARE-mediated function of Nrf2, it may be feasible to impede the neurodegenerative process by suppressing NF-κB’s production of ROS and the subsequent expression of redox-sensitive inflammatory mediators (175, 185).

**Figure 1:**
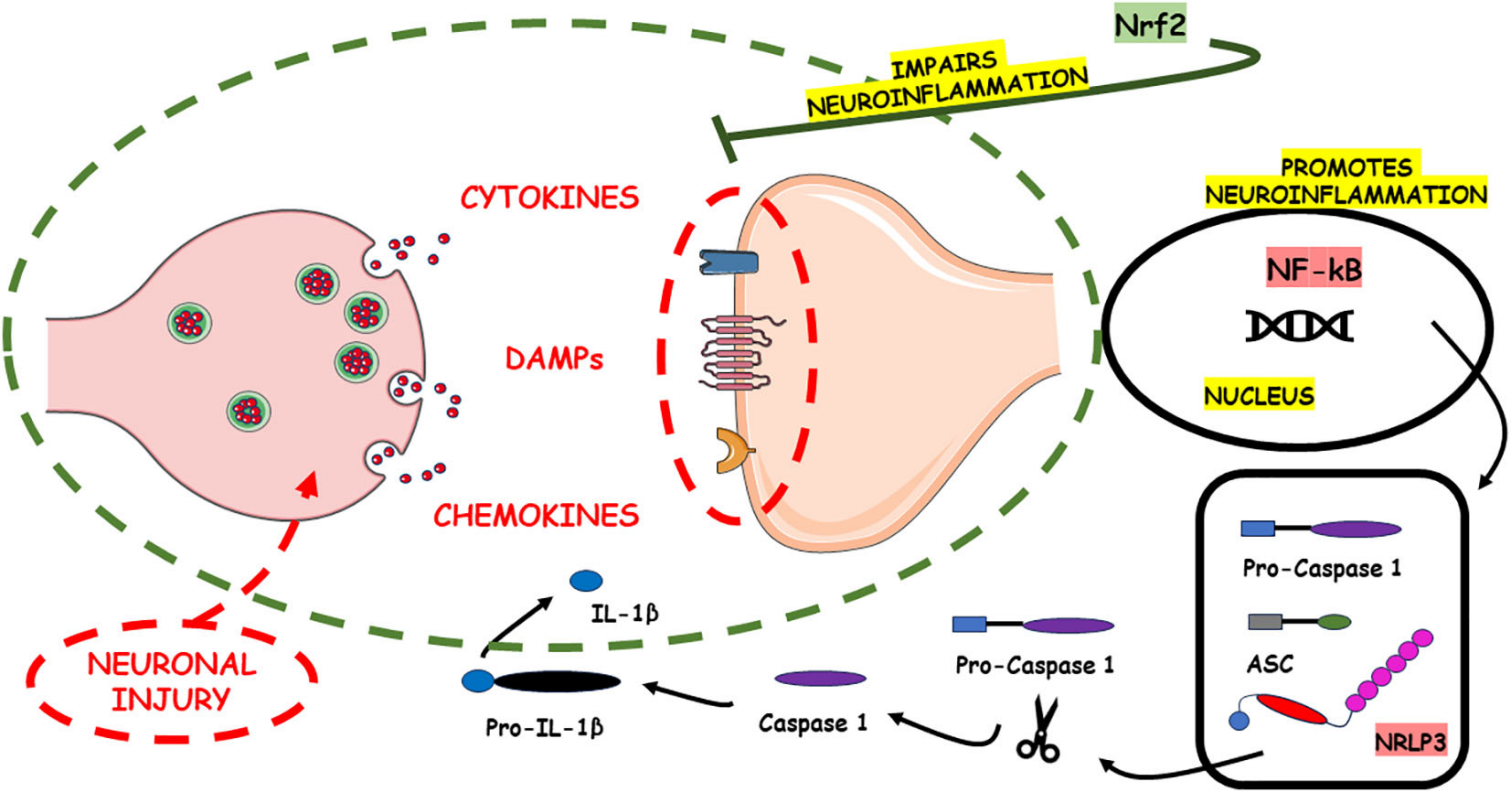
The pro-neuroinflammatory potential associated with the activation of NF-kB and the NLRP3 inflammasome lies in their capacity to initiate and intensify inflammatory cascades within the nervous system. These signaling pathways play pivotal roles in amplifying the inflammatory response. In contrast, Nrf2 emerges as a counteractive force, exerting a neuroinflammatory-suppressing influence. Nrf2 achieves this by finely modulating cellular responses to oxidative stress and inflammation, thus alleviating the adverse consequences commonly associated with neuroinflammation. Through its regulatory actions, Nrf2 serves as a key player in the homeostatic control of inflammatory processes in the nervous system, offering a potential avenue for therapeutic interventions aimed at mitigating neuroinflammatory disorders. The damage to neurons in Alzheimer’s disease is intricately associated with the build-up of irregular protein clusters, including beta-amyloid plaques and tau tangles. These clusters harm synapses and alter neurotransmission, resulting in inflammation and ultimately causing harm to neurons (186). The primary motor symptoms of Parkinson’s disease stem from the loss of dopaminergic neurons in the substantia nigra pars compacta (187). In multiple sclerosis (MS), inflammation is facilitated by various inflammatory cytokines generated by immune cells and local resident cells, including activated microglia. Subsequent harmful processes include the migration of activated B lymphocytes and plasma cells, which produce antibodies targeting the myelin sheath. This amplifies the immune response and ultimately leads to the inflammatory progressive loss of myelin (188).

**Figure 1** illustrates the pro-neuroinflammatory potential of NF-kB and NLRP3 inflammasome activation, alongside the neuroinflammatory-suppressing impact of Nrf2.

## 6 JAK/STAT’s theatrical performance in neuroinflammation and its intriguing connection to the tapestry of neurological disorders

Inflammation can trigger the mobilization of immune cells, initiating the JAK/STAT pathway—a primary signaling route employed by cytokines. This pathway plays a crucial role in kickstarting innate immunity, coordinating adaptive immune mechanisms, and ultimately regulating both inflammatory and immune responses. Over 70 cytokines utilize this pathway, underscoring its significance. Notably, it holds importance in various cancers and neurological disorders. The Janus kinase (JAK) encompasses four isoforms—JAK1, JAK2, JAK3, and TYK2—while signal transducer and activator of transcription (STAT) comprises seven isoforms—STAT1, STAT2, STAT3, STAT4, STAT5A, STAT5B, and STAT6. Structurally, they share five domains: an amino-terminal domain, a carboxy-terminal transactivation domain, an SH2 domain, a DNA-binding domain, and a coiled-coil domain (189).

The STAT proteins were identified as cytoplasmic transcription factors responsible for orchestrating cellular responses to cytokines and growth factors (190). Upon ligand-receptor interaction, STAT activation ensues through the phosphorylation of a critical tyrosine residue in the STAT transactivation domain by various entities, including growth factor receptors, JAKs, SRC family kinases, and other tyrosine kinases (191, 192). This cascade triggers several events, including STAT-STAT dimerization facilitated by a reciprocal phospho-tyrosine (pTyr)-SH2 domain interaction, nuclear translocation, DNA binding, and the transcriptional induction of genes within the nucleus (189).

The conventional (canonical) JAK/STAT signaling pathway entails the interaction of the cell ligand with its receptor, leading to receptor dimerization (193). However, certain receptors like GH receptor, IL-10R, EpoR, IL-17R, TNF-R1, and gp130 can pre-form inactive receptor dimers prior to ligand binding. This pre-formation may facilitate swift assembly of the receptor complex and subsequent signal transduction (Valle-194). The binding of ligand to the receptor induces JAK transphosphorylation (195). Activated JAK, in turn, causes tyrosine phosphorylation of the bound receptor, creating a docking site for STATs (196). At this site, JAK phosphorylates STAT, leading to the dissociation of STAT from the receptor. Subsequently, STAT forms homodimers or heterodimers through SH2-domain–phosphotyrosine interactions (197). These dimers then translocate to the promoters of target genes, regulating their transcription (198). STAT typically regulates transcription through various mechanisms. Firstly, it binds to its DNA target site for transcription activation. Secondly, it forms a transcription complex with non-STAT transcription factors to initiate transcription mediated by STAT. Thirdly, it associates with non-STAT DNA-binding elements to promote STAT-dependent transcription. Fourthly, it synergistically activates transcription with non-STAT transcription factors by binding to clusters of independent DNA-binding sites (199).

Research conducted on Drosophila has revealed an unconventional pathway of JAK–STAT signaling. In contrast to the conventional signaling pathway where the latent STAT protein resides in the cytoplasm, the non-canonical pathway involves a fraction of unphosphorylated STAT localized in the nucleus, specifically on heterochromatin in association with HP1. The unphosphorylated STAT associated with heterochromatin plays a crucial role in preserving HP1 localization and ensuring the stability of heterochromatin. Phosphorylation-induced activation of STAT results in its dispersion from heterochromatin, leading to the displacement of HP1 and subsequent destabilization of heterochromatin. Notably, this process seems independent of the induction of STAT transcriptional target genes (200).

Physiological negative regulators, such as suppressors of cytokine signaling (SOCS) and protein tyrosine phosphatases (PTPs), play a crucial role in downregulating active STAT signaling (201). However, cytokines, by activating the JAK/STAT pathway, significantly influence the development, differentiation, and function of myeloid and lymphoid cells (193). The dysregulation of the JAK/STAT pathway, particularly in the activation and polarization of myeloid cells and T cells toward pathogenic phenotypes, holds pathological implications for neuroinflammatory diseases (199). The activation of microglial inflammation is also significantly impacted by the JAK/STAT pathway. Wang et al. (202) revealed that the compound FPS-ZM1 effectively hinders LPS-induced microglial inflammation by suppressing the JAK/STAT signaling pathway. FPS-ZM1, recognized as an inhibitor of the receptor for advanced glycation end products (RAGE), exhibited the capability to reduce the overproduction of IL-1β, IL-6, TNF-α, and cyclooxygenase-2 (COX-2) induced by LPS, both in BV-2 cells and primary microglial cells. Moreover, FPS-ZM1 ameliorated the proliferation and activation of microglia in the hippocampus of C57BL/6J mice challenged with LPS. Concurrently, the excessive production of pro-inflammatory cytokines IL-1β and TNF-α in the hippocampus was alleviated following FPS-ZM1 treatment. Further investigations revealed that FPS-ZM1 downregulated the LPS-induced increase in phosphorylation levels of JAK/STAT, both *in vivo* and *in vitro*. FPS-ZM1 also demonstrated the ability to inhibit the nuclear translocation of transcription factors STAT1/3/5 in BV-2 cells. Additionally, inhibiting the JAK/STAT signaling pathway independently exhibited anti-inflammatory effects similar to FPS-ZM1 treatment (203).

In AD, Chiba et al. (204) confirmed that the JAK2/STAT3 axis serves as a key mediator of the neuroprotective effects induced by humanin (HN). The Aβ-dependent deactivation of the JAK2/STAT3 axis in hippocampal neurons results in cholinergic dysfunction through both pre- and post-synaptic mechanisms, contributing to memory impairment associated with AD. Emphasizing the significance of an AD-specific neuroprotective peptide HN in mitigating AD-related neurotoxicity by activating the JAK2/STAT3 signaling axis, these researchers observed that the age- and disease-dependent decline in the JAK2/STAT3 axis plays a pivotal role in the pathogenesis of AD. Another correlation is evident in the p-STAT3 immunoreactivity observed in hippocampal neurons of young individuals compared to older normal subjects, both in humans and rodents, and the endogenous levels of insulin-like growth factor-1 activating STAT3. The levels of both p-STAT3 and insulin-like growth factor-1 decrease with aging, suggesting a potential connection to the pathogenesis of AD. Considering that disrupted STAT3 activity due to aging and the neurotoxic effects of amyloid contribute to memory impairment associated with AD, the activation of STAT3 emerges as a novel therapeutic strategy for the condition. The therapeutic mechanisms of STAT3 activation appear to involve the augmentation of cholinergic neurotransmission (205).

Varma et al. (206) documented that hydroxychloroquine (HCQ) reduces the risk of AD and related dementias while also ameliorating molecular phenotypes associated with AD. In a study involving 109,124 rheumatoid arthritis patients receiving routine clinical care, the initiation of HCQ showed a decreased risk of incident AD compared to methotrexate initiation, addressing various biases through four alternative analysis schemes. Additional research further revealed that HCQ has dose-dependent effects on late long-term potentiation (LTP), rescuing impaired hippocampal synaptic plasticity before significant amyloid plaque accumulation and neurodegeneration in APP/PS1 mice. Moreover, HCQ treatment enhanced microglial clearance of Aβ1-42, reduced neuroinflammation, and diminished tau phosphorylation in cell culture-based phenotypic assays. Notably, the study demonstrated that HCQ inactivates STAT3 in microglia, neurons, and astrocytes, suggesting a plausible mechanism associated with its observed effects on AD pathogenesis.

Qin et al. (207) discovered that blocking the JAK/STAT pathway provides protection against α-synuclein-induced neuroinflammation and dopaminergic neurodegeneration. *In vitro*, exposure to α-synuclein activated the JAK/STAT pathway in microglia and macrophages. Treatment with AZD1480, a JAK1/2 inhibitor, effectively restrained α-synuclein-induced major histocompatibility complex class II and inflammatory gene expression by reducing the activation of STAT1 and STAT3 in microglia and macrophages. In their *in vivo* investigations, the researchers employed a rat model of Parkinson’s Disease (PD) induced by viral overexpression of α-synuclein. AZD1480 treatment curbed α-synuclein-induced neuroinflammation by suppressing microglial activation, infiltration of macrophages and CD4(+) T-cells, and the production of proinflammatory cytokines/chemokines. The substantia nigra of rats with α-synuclein overexpression exhibited heightened expression of numerous genes related to cell-cell signaling, nervous system development and function, inflammatory diseases/processes, and neurological diseases. Remarkably, these effects were mitigated upon treatment with AZD1480. Notably, inhibition of the JAK/STAT pathway played a crucial role in preventing the degeneration of dopaminergic neurons *in vivo*. Khera et al. (208) reviewed the preventive roles of modulators targeting JAK-STAT and PPAR-Gamma signaling in autism and neurological dysfunctions. JAK activation induces the phosphorylation of STAT3 in astrocytes and microglia, a process associated with mitochondrial damage, apoptosis, neuroinflammation, reactive astrogliosis, and genetic mutations. Acting as a regulator within the context of JAK-STAT signaling, PPAR-gamma plays a crucial role in preventing such phosphorylation, thereby contributing to the treatment of the mentioned neurological complications.

Shao et al. (209) documented that inhibiting JAK ameliorated experimental autoimmune encephalomyelitis by interrupting the GM-CSF-driven inflammatory characteristics of monocytes. JAK inhibition hindered the infiltration of C-C chemokine receptor type 2 (CCR2)-dependent Ly6Chi monocytes and monocyte-derived dendritic cells into the CNS in experimental autoimmune encephalomyelitis (EAE) mice. Concurrently, JAK inhibition reduced the proportion of GM-CSF+CD4+ T cells and the secretion of GM-CSF in pathological Th17 cells. This, in turn, transformed CNS-invading monocytes into antigen-presenting cells, contributing to the mediation of tissue damage. In a separate *in vivo* investigation, Chen et al. (210) utilized a mouse model of EAE to assess the mitigating effects of magnolol on myeloencephalitis. Using *in vitro* methods, a fluorescence-activated cell sorting (FACS) assay was employed to examine the impact of magnolol on the differentiation of Th17 and Treg cells, as well as the expression of IL-17A. The *in vivo* results demonstrated that magnolol alleviated the loss of body weight and the severity of EAE in mice. It also improved spinal cord lesions, reduced CD45 infiltration, and lowered serum cytokine levels. Correspondingly, magnolol exhibited a focus on inhibiting Th17 differentiation and IL-17A expression in the splenocytes of EAE mice. Furthermore, magnolol selectively inhibited p-STAT3 and p-STAT4 in both CD4+ and CD8+ T cells in the splenocytes of EAE mice. The *in vitro* experiments revealed that magnolol selectively hindered Th17 differentiation and IL-17A expression without affecting Treg cells. A network pharmacology-based study suggested that magnolol might diminish Th17 cell differentiation by regulating STAT family members. Western blotting confirmed that magnolol inhibited p-JAK2 and selectively counteracted p-STAT3 while slightly decreasing p-STAT4. Magnolol also antagonized both the nuclear location and transcription activity of STAT3. High-affinity binding between magnolol and STAT3 was observed, with the specific binding site potentially located at the SH2 domain. Additionally, the overexpression of STAT3 resulted in the failure of magnolol to inhibit IL-17A.

## 7 The dramatic influence of Toll-like receptors on neuroinflammation and their intriguing links to the complex landscape of neurological disorders

TLRs form a crucial receptor family constituting the primary defense line against microbes (211). They can identify invading pathogens as well as endogenous danger molecules released from dying cells and damaged tissues, playing a pivotal role in bridging innate and adaptive immunity (212). TLRs are widely distributed across immune and other body cells, with their expressions and locations regulated in response to specific molecules from pathogens or damaged host cells (213). Ligand binding to TLR activates distinct intracellular signaling cascades, initiating host defense reactions (214). This binding is ligand-dependent and cell type-dependent, resulting in the production of pro-inflammatory cytokines and type 1 interferon (215). The TLR-dependent signaling pathways are rigorously controlled during innate immune responses by various negative regulators (216). Excessive TLR activation can disrupt immune homeostasis, increasing the risk of inflammatory diseases and autoimmune disorders. Consequently, antagonists and inhibitors targeting TLR signaling pathways have emerged as novel therapeutics for the treatment of these diseases (217).

TLRs belong to the category of type I integral transmembrane proteins, typically composed of three domains (218). These include an N-terminal domain (NTD) situated externally to the membrane, a transmembrane domain with a single helix spanning the membrane, and a C-terminal domain (CTD) positioned toward the cytoplasm. The N-terminal domain functions as an ectodomain, serving as the site for ligand recognition of various PAMPs. Meanwhile, the CTD participates in interactions with various signal transduction adaptors, initiating downstream signaling through its toll-IL-1 receptor (TIR) homologous domain (219). The ectodomain exhibits a folded solenoid structure (resembling a horseshoe) containing highly conserved short tandem leucine-rich repeat LRR motifs (220). These LRR motifs, with a sequence pattern of xLxxLxLxx, provide essential specificity and recognizability to TLRs for principally PAMPs (221). Each LRR repeat sequence of the TLRs consists of 24-29 amino acids (222). Additionally, the NTD contains glycan moieties that serve as the actual binding sites for various ligands derived from pathogens (223). The interaction between ligands and TLR initiates specific intracellular downstream signaling cascades, triggering host defense reactions. These interactions between PAMPs and PRRs produce pro-inflammatory cytokines and type 1 interferon, guiding immune responses. TLR signaling is contingent on the stimulus nature, the activated TLR, and the downstream adaptor molecule. There are at least two distinct pathways in TLR signaling: the myeloid differentiation primary response 88 (MyD88)-dependent pathway, which is employed by all TLRs except TLR3, leading to the generation of inflammatory cytokines, and the TIR-domain-containing adapter-inducing interferon (TRIF)-dependent pathway, which is utilized by TLR3 and 4 and is associated with the stimulation of interferon type-1 (213).

TLRs exhibit broad expression within the CNS and fulfill diverse roles in either cell survival or cell death processes. Numerous studies have indicated the presence of TLRs in various cell types within the CNS, including neurons, microglia, astrocytes, oligodendrocytes, and neural stem cells (224). The interaction between ligands and TLR initiates specific intracellular downstream signaling cascades, triggering host defense reactions. These interactions between PAMPs and PRRs produce pro-inflammatory cytokines and type 1 interferon, guiding immune responses. TLR signaling is contingent on the stimulus nature, the activated TLR, and the downstream adaptor molecule. There are at least two distinct pathways in TLR signaling: the MyD88-dependent pathway, which is employed by all TLRs except TLR3, leading to the generation of inflammatory cytokines, and the TRIF-dependent pathway, which is utilized by TLR3 and 4 and is associated with the stimulation of interferon type-1 (213). TLR2 is a prominent member among TLRs within the CNS. An examination of its immunoreactivity in the neurogenic regions of the adult brain has uncovered its presence on cells in both the subgranular zone (SGZ) of the hippocampal dentate gyrus (DG) and the subventricular zone (SVZ) of the lateral ventricles associated with neurogenesis (225).

The activity of TLRs is associated with several neurodegenerative diseases, including stroke, amyotrophic lateral sclerosis (ALS), PD, and AD. TLRs are expressed in various cell types, such as neurons and glia, where they recognize DAMPs released by undifferentiated or necrotic cells (225). Microglia exhibit the expression of all TLRs and their adapter proteins, as evidenced in mice, rats, and humans. This includes TLR1 and TLR6, TLR2, TLR3, TLR5, TLR7, TLR8, TLR4, and TLR9. The constitutive expression of TLRs is primarily observed in microglia and is predominantly confined to the circumventricular organs (CVOs) and meninges—areas with direct access to the circulation. While there may be lower expression levels in other regions, different stimuli, such as hypoxia, LPS, kainic acid, α-synuclein, and Aβ, can lead to increased expression of TLRs in microglia (224). In AD, Aβ triggers the activation of Toll-like receptor 4 (TLR4) in microglia. TLR4 activation initiates downstream signaling pathways, producing these cytokines, which, in turn, activate astrocytes and influence amyloid-dependent neuronal death. Consequently, by modulating neuroinflammation, TLR4 emerges as a significant molecular target for potential AD treatment (226).

Researchers have also contemplated the potential engagement of TLRs in PD. While the exact mechanism by which TLRs contribute to neuroinflammation in PD remains unclear, specific theories suggest their potential role in recognizing α-synuclein aggregates as DAMPs. This recognition could initiate proinflammatory downstream pathways, thereby contributing to the development of neuroinflammation (227). In this scenario, da Silva et al. (228) reported that in PD, there is a deficiency in the immune responses of blood leukocytes to TLR2 and TLR7/8. Twenty-one individuals with PD and 21 healthy controls were enlisted for the study. Patient assessments were conducted using the Unified PD Rating Scale and the Hoehn and Yahr stage. Cytokine levels were quantified in supernatants from whole blood cultures following incubation with TLR2, TLR4, or TLR7/8 agonists using cytometric bead array. Additionally, cytometry was employed to analyze the expression of CD14, CD16, TLR2, and TLR4. Blood cells from patients exhibited reduced cytokine levels in response to TLR2 and TLR7/8/R848 activation compared to controls. The proportions of CD14+CD16+ and CD14+CD16- monocytes and the expression of TLR2 and TLR4 were comparable between patients and controls. The findings were not linked to an imbalance in monocyte subsets or altered TLR2/TLR4 expression in these cells.

TLRs are pivotal in the development of MS. Bsibsi et al. (229) examined the expression of TLR3 and TLR4 through immunohistochemical analysis of brain and spinal cord sections obtained from both control subjects and individuals with multiple sclerosis. The results unveiled heightened expression of TLR in inflamed CNS tissues. Hossain et al. (230) conveyed that the levels of the soluble form of TLR-2 are increased in the serum of individuals with MS, suggesting a potential novel biomarker for the disease. Ferreira et al. (231) indicated that various subsets of TLR-positive T-cells secreting IL-17 are linked to disease activity in MS. Within diverse IL-17+ T-cell profiles, the percentage of IL-17+ TLR+ CD4+ and CD8+ T-cells, generating interferon (IFN)-γ or IL-6, exhibited a positive correlation with both the quantity of active brain lesions and neurological impairments. Furthermore, stimulation of purified CD4+ and CD8+ T-cells with TLR ligands, specifically TLR-2 (Pam3Csk4), TLR-9 (oligodeoxynucleotide [ODN]), and TLR-4 (LPS), directly triggered cytokine production in the patients with MS. Among the various TLR ligands, Pam3Csk4 demonstrated greater potency than other TLR ligands in inducing the production of proinflammatory cytokines. Additionally, levels of IL-6, IFN-γ, IL-17, and granulocyte-macrophage colony-stimulating factor (GM-CSF) produced by Pam3Csk4-activated CD4+ cells were directly correlated with disease activity. Nyirenda et al. (232) reported that stimulation of TLR2 regulates the equilibrium between regulatory T cell and Th17 function, presenting a novel mechanism for the diminished regulatory T cell function in MS patients. CD4(+)CD25(hi) FOXP3(+) regulatory T cells (Tregs) play a crucial role in maintaining self-antigen tolerance, and their impaired function contributes to the pathogenesis of MS. In this experiment, Tregs derived from MS patients exhibit elevated levels of TLR2 compared to those from healthy individuals. Stimulation with the synthetic lipopeptide Pam3Cys, an agonist of TLR1/2, diminished Treg function and promoted Th17 skewing more significantly in MS patient samples than in healthy controls.

## 8 Navigating the course of neuroinflammation and microglial activation: insights into multiple sclerosis, Alzheimer’s disease, and Parkinson’s disease

Microglia, crucial cells within the CNS, have their origins in the yolk sac and undergo a differentiation process regulated by interferon regulatory factor 8 (IRF8) and the transcription factor PU.1 (233). Their continued existence relies on the signaling of the colony-stimulating factor 1 (CSF1) receptor (234). These microglial cells are pivotal in CNS development and regulating higher cognitive functions. They also maintain the balance of the CNS microenvironment by engulfing deceased cells, cellular debris, and misfolded proteins (235). Besides, proliferating reactive microglia gather in areas characterized by elevated concentrations of apoptotic neurons, functioning as phagocytes to facilitate neuronal turnover in the context of developmental cell death. Additionally, they play a role in regulating synaptic function (236).

Microglia and macrophages are categorized into two distinct phenotypes with opposing functions: M1 and M2 (237). M1 microglia predominantly stimulates the production of nitric oxide synthase 2 (iNOS/NOS2) through upregulating interferon-γ. Consequently, this correlates with the generation of nitric oxide (NO) (238). Additionally, they release various chemokines and inflammatory factors (IL-12, IL-6, IL-1β, CCL2, TNF-α) through the MAPK and NF-κB pathways. They also express costimulatory molecules (CD36, CD47, CD45), integrins (CD11b, CD11c), major histocompatibility complex II (MHC II), and Fc receptors (235, 239). Conversely, M2 microglia secrete an array of anti-inflammatory factors, such as IL-4 and IL-13, along with growth factors and neurotrophic factors like glial cell-derived neurotrophic factor (GDNF) and brain-derived neurotrophic factor (BDNF). These factors aid in alleviating neuro-inflammatory responses and provide overall neuroprotection (240–242).

Besides, recent findings offer evidence for the presence of potential microglial subtypes characterized by distinct genomic, spatial, morphological, and functional features. While microglia are distributed ubiquitously throughout the CNS, their presence varies across regions and between the white and gray matter. The morphology of microglia differs based on the proximity of neuronal cell bodies, dendrites, axons, myelinated axons, and blood vessels. Additionally, under normal physiological conditions and in response to stimuli such as LPS challenge, microglia exhibit regional variations in self-renewal and turnover rates. It has been demonstrated that the regional microenvironment plays a crucial role in determining microglial identity at the transcriptional level in both mice and humans (243). Aligned with the pronounced microglia’s spatial organization observed in the CNS, the majority of CNS diseases exhibit a distinct regional distribution. While microglia have been recognized as crucial contributors to various CNS diseases, it remains uncertain whether microglia exhibit properties specific to particular regions in the context of neurodegenerative diseases.

Several investigations have explored the polarization responses of microglia concerning aging. Recent findings revealed an age-dependent increase in transcript and protein levels of M1 markers (TNFα and IL-1β) and a contrasting trend in the expression of M2 markers (Arg1 and IL-10). This observed rise in the M1/M2 marker ratio also correlated with age-related dopaminergic (DA) neuronal loss. Additionally, a decline in the M2-like phenotype, characterized by suppressed anti-inflammatory IL-4/IL-13 signaling, was observed in aging mice. Aging-related changes were further underscored by increased M1-like microglial responses, including the upregulation of TLRs, various activation markers (MHCII, CD68, and CD86), and inflammatory receptors specific to microglia/macrophages such as CD11b. These alterations are evident not only in the brains of aged rodents but also in canines, humans, and non-human primates. These findings suggest that microglia in aging individuals tend to exhibit a predominant M1-like phenotype associated with neurotoxic responses (244, 245).

The presence of reactive microglia in the aged brain has also been linked to the age-related decline in inherent regulatory pathways within microglia. Zöller et al. (246) provided evidence that the silencing of transforming growth factor β (TGFβ) signaling in microglia leads to impaired homeostasis. Interestingly, the deletion of transforming growth factor, beta receptor 2 (Tgfbr2) in adult postnatal microglia does not result in the impairment of microglia-specific gene expression signatures, nor does it affect microglial survival and maintenance. However, Tgfbr2-deficient microglia exhibit distinctive morphological changes. Transcriptome analysis using RNAseq revealed that the loss of TGFβ signaling leads to the upregulation of markers associated with microglial activation and priming. Additionally, protein arrays demonstrated increased secretion of C-X-C motif chemokine ligand 10 (CXCL10) and C-C motif chemokine ligand 2 (CCL2), accompanied by the activation of immune cell signaling, as evidenced by an increase in the phosphorylation of tat-associated kinase 1 (TAK1). In a similar context, Tichauer et al. (247) noted that the activation of the TGFβ1-Smad3 pathway is compromised during the aging process. The authors also highlighted that the age-related dysfunction in the TGFβ1-Smad3 pathway may diminish protective activation while promoting cytotoxic activation of microglia, thereby intensifying microglia-mediated neurodegeneration. TGFβ1 influenced the stimulation of NO and ROS production in young and adult microglia, respectively. This modulation was, to some extent, reliant on the mothers against decapentaplegic homolog 3 (Smad3) pathway and was hindered by inflammatory preconditioning. In microglia cultures from young mice, inflammation and TGFβ1 prompted phagocytosis, while TGFβ1-induced phagocytosis was also hindered by Smad3 inhibition.

Hammond et al. (248) conducted single-cell RNA sequencing on microglia across the lifespan of mice and in the context of brain injury, revealing intricate changes in cell states. Their primary discovery involved identifying a subset of microglia characterized by the selective expression of the chemokine Ccl4. These microglia were initially present in limited numbers during development but undergo expansion in two specific contexts: aging and injury. While the role of inflammatory molecules in the brain has been extensively studied, with previous assumptions based largely on *in vitro* research suggesting microglia as a major source of these factors, the researchers found that the only microglia enriched for inflammatory signals belonged to Cluster 8/Cluster OA2/Cluster IR2.2, IR2.3. This subset expressed Ccl3, Ccl2, Ccl7, Ccl9, Ccl12, Il1b, and Tnf. Given the rarity of this small subpopulation throughout the mouse lifespan, it is plausible that they constitute a specialized group uniquely primed for generating an inflammatory response. Notably, many of the signals expressed in this subpopulation have the potential to cause significant damage to the brain.

Griciuc et al. (249) revealed that the receptors CD33 and triggering receptor expressed on myeloid cells 2 (TREM2) in microglia have been linked to AD risk. Investigating the interplay between CD33 and TREM2, the researchers found that knocking out CD33 mitigated Aβ pathology and enhanced cognition in 5xFAD mice. However, these improvements were negated by additional knockout of TREM2. Conversely, when TREM2 was knocked out in 5xFAD mice, it exacerbated Aβ pathology and neurodegeneration but reduced the numbers of Iba1+ cells. Importantly, the additional knockout of CD33 did not rescue these effects. RNA-seq profiling of microglia indicated an upregulation of genes related to phagocytosis and signaling (IL-6, IL-8, acute phase response) in 5xFAD;CD33^-/-^ mice and a downregulation in 5xFAD;TREM2^-/-^ mice. The differential gene expression in 5xFAD;CD33^-/-^ microglia was dependent on the presence of TREM2, suggesting that TREM2 acts downstream of CD33.

In Pulido-Salgado et al.’s (250) experiment, murine primary microglial cultures were subjected to a 6-hour treatment with LPS or LPS + IFNγ, followed by RNA-Sequencing analysis. Utilizing weighted gene co-expression network analysis (WGCNA), the researchers identified 11 distinct expression profiles that revealed varied responses to LPS and LPS + IFNγ across numerous genes. Notably, a subset of genes associated with PD, AD, and Huntington’s disease exhibited downregulation under both treatments. DESeq analysis further confirmed LPS and LPS + IFNγ as inducers of microglial pro-inflammatory responses while highlighting their involvement in specific cellular functions. Under LPS treatment, microglia demonstrated a propensity for increased proliferation, pro-inflammatory activity, and phagocytosis. Conversely, LPS + IFNγ treatment led to the inhibition of genes associated with pain, cell division, and unexpectedly, the production of certain inflammatory mediators. In summary, this study provides a comprehensive exploration of the transcriptome of primary microglial cultures treated with LPS and LPS + IFNγ, offering insights into their distinct effects on microglial gene expression and cellular functions.

Through comprehensive RNA-seq analysis encompassing AD, ALS, and aging, a novel and infrequent microglial subset known as Disease-Associated Microglia (DAM) was identified and found to be conserved in both mice and humans. Molecularly, DAM are characterized as immune cells expressing conventional microglial markers, namely Iba1, Hexb, and Cst3, concomitant with the downregulation of “homeostatic” microglial genes, including purinergic receptor P2Y (P2ry) 12, CX3C motif chemokine receptor 1 (Cx3cr1), transmembrane protein 119 (Tmem119), CD33, and P2ry13. Additionally, DAM exhibit an upregulation of genes associated with lysosomal, phagocytic, and lipid metabolism pathways, including several well-known AD risk factors such as cathepsin D (CtsD), Trem2, TYRO protein tyrosine kinase-binding protein (Tyrobp), lipoprotein lipase (Lpl), and apolipoprotein E (ApoE). Initially identified in a mouse model of AD expressing five human familial AD mutations (5XFAD), DAM characteristics have been subsequently validated in other Aβ AD mouse models, including PS2APP and APP/PS1. Crucially, DAM were primarily observed in CNS regions affected by the disease and not in other unaffected regions (251). Moreover, the analysis of Trem2^–/–^ × 5XFAD mice revealed that the transformation of homeostatic microglia into DAM is a gradual process occurring through two sequential yet distinct stages. The first stage, termed DAM1, is TREM2-independent and involves the activation of Tyrobp, B2-microglobulin (B2m), and Apoe, along with the downregulation of microglia checkpoint genes (such as Cx3cr1 and P2ry12/P2ry13). Subsequently, the second stage, DAM2, is TREM2-dependent and includes the upregulation of phagocytic and lipid metabolism genes (such as CD9, Lpl, and Cst7) (252).

Two hypotheses have been proposed regarding how TREM2 signaling contributes to the phenotypic transition from stage 1 to stage 2 DAM. First, TREM2 may uphold the activation of microglia induced by other receptors during stage 1. Second, TREM2 signaling could kickstart a transcriptional program specific to stage 2. Recent data lean toward the first hypothesis, indicating that TREM2’s pro-proliferative and pro-survival functions through phosphoinositide 3-kinase (PI-3K), β-catenin, and mechanistic target of rapamycin (mTOR) pathways suggest its role in sustaining microglial activation and survival, rather than triggering additional transcriptional programs. However, further studies are necessary to unravel the precise mechanisms governing DAM regulation (251, 253–255).

MS is a noteworthy neurodegenerative and neuroinflammatory condition that demands attention. In the initial stages of MS development, roughly 40% of the initial phagocytic cells are microglia, identifiable by the TMEM119 marker, specific to microglia and distinct from macrophages (256). As the lesion progresses, peripheral macrophages are recruited more frequently (257). Within an active lesion, virtually none of the microglial cells are in a homeostatic state, as evidenced by the absence of P2RY12, a receptor unique to the ramified microglial processes seen in the resting state (258–260). Clusters of activated microglia are present in the typical white matter of MS patients (261). However, previous studies have shown conflicting results regarding the expression of P2RY12 in these clusters (260, 262). This leads to the conclusion that the heterogeneity of microglial phenotypes, previously observed in mice, is also apparent in progressive MS patients, with upregulated genes involved in lipid processing in the white matter and iron homeostasis in the gray matter (263). The metabolic alterations in microglia reflect MS physiopathology, even in the absence of demyelinating lesions, underscoring the distinct inflammatory processes in white and gray matter in MS (264, 265). Indeed, active demyelination is associated with a proinflammatory microglia phenotype, characterized by p22phox, CD68, CD86, and MHC II antigens. In contrast, anti-inflammatory markers like CD206, CD163, and ferritin are most prominent in the center of inactive lesions (266, 267).

Conversely, microglia’s internalization of myelin in various animal models triggers a pro-regenerative phenotype marked by arginase-1, CD206, and insulin-like growth factor-1 (IGF-1), facilitating oligodendrocyte differentiation and remyelination (268, 269). Objectively, the average human brain microglia exhibit an intermediate activation state (presence of CD68 and reduced P2RY12), differing from the homeostatic state seen in animal models (256, 270). This implies potential variations in microglial behavior between humans and other species, which may be attributed to higher levels of systemic inflammation (260). Recent research emphasizes that demyelination is an even more intricate scenario than previous studies indicated. For instance, contemporary literature underscores the potential of microglia in regulating BMP-4, which might impede remyelination, and its antagonist, noggin, which is more highly expressed in areas of remyelinated lesions (271–273). Recent discoveries indicate that genes associated with MS susceptibility are more frequently linked to microglial function than neurons or astrocytes, signifying a significant role for microglia in MS pathogenesis (274). However, mutations in the CSF1-receptor (CSF1R) gene, a pivotal microglial-specific gene associated with other leukoencephalopathies, have not been correlated with MS pathology, as no relevant mutations were identified in CSF1R sequencing studies of MS patients (275–278).

In AD, the most prevalent form of dementia, cognitive dysfunction arises not directly from Aβ, but rather from the accumulation of Tau and alterations in synaptic density within the hippocampus (279). However, emerging research suggests that soluble ß oligomers in AD’s pathophysiology interact with neuronal synapses, modifying their structure, composition, and density. This leads to cognitive impairments and enduring neurological deficits (186, 280). Despite progress in understanding this intricate scenario in recent years, the exact roles of microglia in AD remain partially elucidated (281). Studies using animal models of AD have demonstrated that microglial cells undergo changes in the early stages and progression of the disease, including diminished branching and protrusion, along with heightened expression of proinflammatory factors (282–285). This compromises the microglia’s capacity to survey the neuronal environment, causing them to shrink gradually, resulting in severe impairments and a decline in cognitive function (235).

Conversely, while the general risk of inflammatory diseases may not heavily impact age-related cognitive decline, functional susceptibility factors such as genetics seem to exert a more substantial influence. They play a role in determining the quantity of microglia during the aging process of the nervous system’s immune system and in regulating the expression of immune-inflammatory genes within cells of the central nervous system (286–288). Several other factors may be associated with microglia activation in the context of the progression of AD. In cases of late-onset AD, a notable surge in progranulin (PGRN) levels in cerebrospinal fluid has been linked to severe cognitive decline. This elevation in PGRN is correlated with heightened levels of soluble TREM2 (sTREM2) in cerebrospinal fluid, suggesting a potential involvement of serum sTREM2 in microglial activation (289, 290). Notably, the absence or reduction of receptors like signal regulatory protein α (SIRPα) and cannabinoid receptor 1 (CB1) on microglia, as well as the receptor CD2000 on neurons, can lead to an increased loss of synapses and cognitive impairment mediated by microglia and phagocytosis (291–294). Additionally, fibrin derived from the bloodstream can harm synapses through the CD11b/CD18 receptors on microglia, contributing to cognitive impairment through CD11b/CD18-mediated microglial activation (194, 295).

Autophagy, a cellular process conserved throughout evolution and dependent on lysosomes in eukaryotes, is intricately connected to regulating protein metabolism. This mechanism involves degrading and subsequently recycling damaged organelles and misfolded proteins to preserve protein homeostasis. Increasing evidence indicates that compromised autophagy plays a role in contributing to the development of AD (241). Autophagy is a pivotal regulator in generating and clearing Aβ (296). Aβ peptides form during the autophagic turnover of amyloid precursor protein (APP) within autophagosomes, where APP undergoes cleavage (297). In AD, impediments arise in the maturation of autophagolysosomes—autophagosomes that have fused with lysosomes—and their retrograde passage toward the neuronal body. This hindrance leads to a significant accumulation of autophagic vacuoles in neurons, potentially linked to dysfunction in the endosomal sorting complexes for transport-III (ESCRT-III) complex. Such malfunction is associated with neurodegeneration and can disrupt the fusion of autophagosomes with the endolysosomal system, impacting autophagosome maturation. Two pathways exist for the disposal of Aβ peptides. Firstly, they can undergo degradation facilitated by various Aβ-degrading proteases, including beta-secretase 1 (BACE1) and CTSD. Secondly, Aβ peptides may amass in autophagosomes within dystrophic neurites, the primary constituents of neuritic senile plaques in AD, becoming integrated into the primary intracellular reservoir of toxic peptides. This latter recycling path of Aβ peptides is particularly prevalent in the brains of individuals afflicted with AD (298).

Luo et al. (299) observed that activating peroxisome proliferator-activated receptor alpha (PPARA)-mediated autophagy effectively reduces AD-like pathology and cognitive decline in a murine model. PPARA, also known as PPARα, serves as a transcription factor regulating genes involved in fatty acid metabolism and hepatic autophagy activation. The study revealed that pharmacologically activating PPARA with the agonists gemfibrozil and Wy14643 induces autophagy in human microglia cells and U251 human glioma cells expressing the human APP mutant (APP-p.M671L), and this induction is PPARA-dependent. The administration of PPARA agonists resulted in decreased amyloid pathology and the reversal of memory deficits and anxiety symptoms in APP-PSEN1ΔE9 mice. There was a notable reduction in soluble Aβ and insoluble Aβ levels in hippocampus and cortex tissues from APP-PSEN1ΔE9 mice after treatment with either gemfibrozil or Wy14643. Additionally, this treatment promoted the recruitment of microglia and astrocytes to the vicinity of Aβ plaques and enhanced autophagosome biogenesis.

Wani et al. (300) documented that crocetin, a bioactive component derived from the flower stigmas of *Crocus sativus*, facilitates the elimination of Aβ by triggering autophagy through the serine/threonine kinase 11 (STK11)/liver kinase B1 (LKB1)-mediated adenosine monophosphate-activated protein kinase (AMPK) pathway. Furthermore, crocetin exhibited the ability to traverse the blood-brain barrier and induce autophagy in the hippocampi of the brains of wild-type male C57BL/6 mice. Subsequent investigations in transgenic male 5XFAD mice, serving as an AD model, also revealed that a one-month treatment with crocetin significantly diminished Aβ levels and mitigated neuroinflammation in the mice brains. This treatment also improved memory function by triggering autophagy mediated by the activation of the AMPK pathway.

Chen et al. (301) discovered that alleviating AD is possible by inhibiting miR-331-3p and miR-9-5p to enhance autophagy. The biphasic alterations in microRNA levels were derived from RNA-seq data and validated through real time quantitative polymerase chain reaction (qRT-PCR) in early-stage (6 months) and late-stage (12 months) APPswe/PS1dE9 mice, referred to as AD mice. AD progression was assessed by analyzing Aβ levels, neuron numbers, and activated microglia (CD68+IBA1+) in brain tissues using immunohistological and immunofluorescent staining. The autophagic activity was determined by testing messenger RNA (mRNA) and protein levels of autophagy-associated genes (Becn1, Sqstm1, and LC3b). Morris water maze and object location tests were employed to evaluate memory and learning post-antagomir treatments in AD mice, with assessed Aβ levels in brain tissues. MiR-331-3p and miR-9-5p were found to be down-regulated in the early stage of AD mice and up-regulated in the late stage. The study demonstrated that miR-331-3p and miR-9-5p target autophagy receptors Sqstm1 and Optineurin Optn, respectively. Overexpression of these microRNAs in the SH-SY5Y cell line impaired autophagic activity and promoted the formation of amyloid plaques. Furthermore, late-stage treatment of AD mice with miR-331-3p and miR-9-5p antagomirs resulted in enhanced Aβ clearance, improved cognition, and increased mobility.

Besides, several studies have underscored the crucial involvement of dysregulated metabolism in the pathogenesis of AD. Metabolic changes in the aging brain, including dysregulation in glucose metabolism, dysfunction in glycolysis, disruptions in the tricarboxylic acid cycle, deficits in oxidative phosphorylation, and impairment in the pentose phosphate pathway (PPP), may be interconnected with the development of AD (302). Demarest et al. (303) employed a cross-species methodology to assess conserved metabolic modifications in the serum and brain of human subjects with AD, two AD mouse models, a human cell line, and two *Caenorhabditis elegans* strains with AD characteristics. The authors identified a specific impairment in mitochondrial complex I in cortical synaptic brain mitochondria of female AD mice, while no such impairment was observed in male AD mice. In the hippocampus, synaptic complex I impairment occurred in male and female mice with Polβ haploinsufficiency, underscoring the essential role of DNA repair in mitochondrial function. In non-synaptic, glial-enriched mitochondria from the cortex and hippocampus, there was an increase in complex II-dependent respiration in female AD mice but not in males. These findings suggested a glial upregulation of fatty acid metabolism, potentially compensating for neuronal glucose hypometabolism in AD. Utilizing an unbiased metabolomics approach, the study consistently identified systemic and brain metabolic remodeling evidence, indicating a transition from glucose to lipid metabolism in human AD subjects and AD mice. The authors also established that this metabolic shift is crucial for survival in *C. elegans* and human cell culture AD models. One of the most notable metabolite changes observed in AD was the accumulation of glucose-6-phosphate, an hexokinase inhibitor and a rate-limiting metabolite for the PPP.

Tang et al. (304) also validated the neuroprotective role of glucose-6-phosphate dehydrogenase (G6PD) and the pentose phosphate pathway. G6PD, the crucial enzyme that directs glucose breakdown from glycolysis into the PPP, is vital in generating reduced nicotinamide adenine dinucleotide phosphate (NADPH) within cells. NADPH, in turn, serves as a substrate for glutathione reductase, facilitating the conversion of oxidized glutathione disulfide to sulfhydryl glutathione. Widely recognized for its involvement in inherited deficiencies leading to acute hemolytic anemia triggered by heightened oxidative stress from food or medication, G6PD, and PPP activation have been linked to neuroprotection. Studies conducted in *Drosophila* models revealed that an increased PPP flux due to G6PD induction resulted in enhanced proteostasis and extended lifespan, a phenomenon replicated by transgenic overexpression of G6PD in neurons. Additionally, moderate transgenic expression of G6PD demonstrated improvements in health span in mice. In summary, Increasing G6PD levels offers the advantage of autonomous NADPH production within cells, ensuring the preservation of neurons’ glutathione (GSH) levels. This mechanism serves as a protective measure against both acute and chronic oxidative stresses. Thus, the activation of G6PD, coupled with selective inhibition of NADPH oxidase (NOX) activity, holds promise as a viable neuroprotective strategy for addressing brain injury, diseases, and aging.

Epis et al. (305) reported on the modulatory impact of acetyl-L-carnitine (ALC) on APP metabolism in hippocampal neurons. Despite demonstrating positive effects on AD in double-blind controlled studies, the precise mechanisms underlying its neuroprotective properties remained incompletely understood. This investigation delved into the influence of acetyl-L-carnitine on APP metabolism using *in vitro* models, including a neuroblastoma cell line and primary hippocampal cultures. The study revealed that ALC treatment enhanced alpha-secretase activity and promoted physiological APP metabolism. Notably, ALC facilitated the delivery of disintegrin and metalloproteinase domain-containing protein 10 (ADAM10), a disintegrin, to the post-synaptic compartment, thereby positively modulating its enzymatic activity toward APP. Consequently, ALC has the potential to directly impact the pivotal event in AD pathogenesis, the Aβ cascade, by fostering alpha-secretase activity and influencing the release of the non-amyloidogenic metabolite.

Bielarczyk et al. (306) documented that AβPP-transgenic 2576 mice mimic cell type-specific aspects of acetyl-coa-linked metabolic deficiencies in AD. In the brains of aged Tg2576 mice (14-16 months old), amyloid-β1-42 levels reached 0.6 μmol/kg. The activities of the pyruvate dehydrogenase complex, choline acetyltransferase, and various enzymes involved in acetyl-CoA and energy metabolism remained unchanged in both forebrain mitochondria and synaptosomes of Tg2576 mice, suggesting the preservation of structural integrity, particularly in cholinergic neuronal cells. Nevertheless, transgenic brain synaptosomes exhibited a 25-40% decrease in pyruvate utilization, mitochondrial and cytoplasmic acetyl-CoA levels, acetylcholine content, and quantal release. Conversely, whole brain mitochondria in transgenic mice showed activation of pyruvate utilization, with no alterations in acetyl-CoA content, citrate, or α-ketoglutarate accumulation. These findings indicate that Aβ-induced deficits in acetyl-CoA are localized to mitochondrial and cytoplasmic compartments of Tg2576 nerve terminals, serving as early primary signals in the progression of neurodegeneration. Conversely, acetyl-CoA synthesis in the mitochondrial compartments of glial cells appears to be activated despite accumulated Aβ in transgenic brains.

Microglia use glycolysis and oxidative phosphorylation (OXPHOS) to meet their energy demands. In their resting state, microglia predominantly rely on OXPHOS to generate ATP. However, microglia undergo a metabolic shift upon activation, transitioning from OXPHOS to glycolysis. This alteration in metabolic phenotype has been observed in different neurodegenerative disorders, including PD and AD. Recent investigations have revealed an augmented glucose uptake in microglia in AD in both AD mice and patients. This metabolic state is positively correlated with the degree of neuroinflammation. Moreover, a significant elevation in lactate levels within microglia derived from a transgenic AD mouse model (5XFAD) was observed. This finding suggests that the intensified glycolytic activity in microglia contributes to the disruption of lactate metabolism in AD (307). Following the initial identification of heightened histone lactylation in brain samples from both 5XFAD mice and individuals with AD, Pan et al. (308) observed an elevation in H4K12la levels, specifically within microglia adjacent to Aβ plaques. This lactate-dependent modification of histones is concentrated at the promoters of glycolytic genes, activating transcription and consequently amplifying glycolytic activity. Ultimately, this creates a positive feedback loop involving glycolysis, H4K12la, and PKM2, exacerbating microglial dysfunction in AD. Pharmacological inhibition of PKM2 was found to mitigate microglial activation, and targeted removal of Pkm2 in microglia improved spatial learning and memory in AD mice.

Microglia exhibit a range of ion channels, encompassing sodium, chloride, calcium, potassium, and proton channels. The array of channels found on microglia contributes to the dynamic characteristics of these immune cells within the brain. These ion channels are pivotal in governing various functions of microglia, including proliferation, chemotaxis, phagocytosis, antigen recognition and presentation, apoptosis, and cell signaling, ultimately influencing processes such as inflammation and other essential functions (309). Particularly, disruptions in calcium homeostasis occur during the process of human aging. Recent studies indicated that dense plaques can induce functional changes in calcium signals in mice afflicted with AD (310). Calcium is crucial in numerous signaling pathways and cellular functions within the human body. In the nervous system, voltage-gated calcium channels, specifically L-type calcium channels (LTCCs), are pivotal in neurotransmitter release, synaptic integration, and plasticity. The intricate structure and functional complexity of LTCCs make it challenging to understand their contribution to AD fully. Nevertheless, LTCCs dysfunction is highly implicated in AD occurrence (311).

LTCCs represent the largest category of voltage-gated calcium channels (312). Comprising four distinct pore-forming α1 subunits—Cav1.1 (α1S), Cav1.2 (α1C), Cav1.3 (α1D), and Cav1.4 (α1F)—LTCCs are accompanied by auxiliary subunits α2-δ, β, and γ (313, 314). The Cav1.1 isoform, encoded by the CACNA1S gene, is present in skeletal muscle, contributing to excitation–contraction coupling (315). The Cav1.4 isoform, coded by the CACNA1F gene, is in the retina and plays a role in photoreceptor transmitter release (316). Mutations in Cav1.4 are linked to night blindness (317). Predominantly expressed in the heart and brain, the Cav1.2 and Cav1.3 isoforms have been implicated in disorders related to the nervous system, such as autism, bipolar disorder, and Timothy’s Syndrome. The Cav1.3 isoform, encoded by the CACNA1D gene, is found in the neuroendocrine system, neurons, cochlea, and cardiac pacemaker cells, contributing to functions like cardiac pacemaking, synaptic regulation, excitation-transcription coupling, hearing, and hormone release. Lastly, the Cav1.2 isoform, encoded by the CACNA1C gene on chromosome 12p13, is present in the heart, endocrine system, and neurons. Cav1.2 plays a crucial role in various processes in the nervous system, including activating calcium-dependent ions, enzymes, and potassium channels (311). The transition of microglia from a resting quiescent state to an “activated” immune-effector state is controlled by intracellular Ca2+. Consequently, manipulating intracellular Ca2+ is a valuable approach to influence the microglia phenotype. In this context, antagonists of LTCCs can potentially decrease the release of proinflammatory cytokines and ROS from activated microglia, offering a possible therapeutic avenue for resolving chronic neuroinflammation observed in conditions such as AD and PD (318).

Furthermore, Cheng et al. (319) documented an elevation in the expression of calcium homeostasis modulator family protein 2 (Calhm2) in a mouse model of AD. In 5×FAD mice harboring five familial AD gene mutations, the conventional knockout (KO) of Calhm2 and the conditional microglial KO of Calhm2 demonstrated a significant decrease in Aβ deposition, neuroinflammation, and cognitive impairments. Mechanistically, the absence of Calhm2 suppressed microglial proinflammatory activity while enhancing phagocytic activity, thereby restoring the balance between inflammation and phagocytosis. Additionally, Calhm2 KO reduced acute LPS-induced neuroinflammation.

In recent research, there has been substantial evidence supporting the involvement of microglial pro-inflammatory signaling in the development of PD (320, 321). These studies specifically emphasize the importance of activating the microglial NLRP3 inflammasome in the onset of PD (322, 323). Furthermore, a recent investigation has unveiled intriguing insights into how α-synuclein (α-syn) is regulated within microglia, pinpointing Fyn kinase as the key regulator in this process (324, 325). As a result, researchers believe that Fyn kinase plays a pivotal role in both initiating and driving the activation of the microglial NLRP3 inflammasome in PD (2, 326). This connection has been further supported by *in vitro* analyzes of human microglial response to fibrillar α-syn (327, 328).

Reactive microglia, particularly through TLR2 activation, have been implicated in the transmission of α-syn in PD, as evidenced by the TLR2-dependent uptake of α-synuclein-containing plasma exosomes by microglia (329–331). Additionally, studies have shown that neurons efficiently internalize human α-syn released by microglia previously exposed to PD plasma exosomes, indicating their role as mediators in the spread of α-syn (330, 332, 333).

Besides to their pro-inflammatory signaling in PD and related disorders, microglia play a crucial role in the clearance of α-syn (334–336). A recent study revealed that complement receptor (CR) 4 selectively binds to fibrillary α-syn, triggering the formation of phagolysosomes and facilitating the clearance of this pathological protein (337). Other studies have also suggested that TLR4 initiates an inflammatory response to α-syn and is pivotal in the phagocytic activity and clearance of α-syn by microglia (338).

The involvement of microglial TLR4 was further assessed in a mouse model of prodromal PD. The observed acceleration of pathology in this model, in the context of TLR4 deficiency, provided additional evidence for TLR4’s role in α-syn processing by microglia. This study demonstrated that TLR4 deficiency *in vivo* promoted the spread of α-syn seeds, linked to reduced lysosomal activity in microglia, ultimately leading to accelerated neurodegeneration in the nigral region (339). Moreover, laboratory-based examinations underscored the impact of extracellular α-synuclein in impeding microglial autophagy function. The hindrance of autophagy by α-synuclein seemed to be mediated through TLR4-dependent activation of the p38 and Akt-mTOR signaling pathways (340).

The impact of Calhm2 deletion has also been observed in the context of PD. Bo et al. (341) observed that both conventional KO of Calhm2 and microglial KO of Calhm2 led to a significant reduction in dopaminergic neuronal loss and a decrease in microglial numbers, consequently improving locomotor performance in mice modeling PD. Mechanistically, it was discovered that Calhm2 interacted with EFhd2, regulating downstream STAT3 signaling in microglia. Besides, the knockdown of Calhm2 or EFhd2 suppressed downstream signaling through STAT3 and reduced levels of inflammatory cytokines in microglia. However, Wang et al. (342) also reported that blocking the microglial Cav1.2 Ca2+ channel exacerbates symptoms in a PD model. They demonstrated the expression of Cav1.2 channels in microglia and found that calcium antagonists amplified the neuroinflammatory M1 transition while inhibiting the neuroprotective M2 transition of microglia *in vitro*. Additionally, they observed extensive degeneration of dopaminergic neurons and associated behavioral deficits in microglia-specific Cav1.2 knockdown mice treated with MPTP, a neurotoxin-inducing PD-like symptoms. These findings suggest the detrimental effects of microglial Cav1.2 blockade on PD.

Disrupted autophagy is also a contributing mechanism influencing microglia in the onset and progression of PD. Qin et al. (343) documented that compromised autophagy in microglia exacerbates dopaminergic neurodegeneration by modulating NLRP3 inflammasome activation in experimental models of PD. In a laboratory setting, BV2 microglial cells underwent exposure to LPS with or without autophagy-related gene 5 (Atg5) small interference RNA (Atg5-siRNA). For the *in vivo* study, microglial Atg5 conditional KO (Atg5^flox/flox^; CX3CR1-Cre) mice and their wild-type littermates (Atg5^flox/flox^) received intraperitoneal injections of MPTP for induction of an experimental PD model. These findings demonstrated that the disruption of autophagy by Atg5-siRNA heightened LPS-induced inflammatory responses in BV2 cells and led to increased apoptosis in SH-SY5Y cells treated with BV2 conditioned medium. In mice, impaired autophagy in microglia worsened dopaminergic neuron loss in response to MPTP. The mechanism underlying the promotion of neuroinflammation and dopaminergic neurodegeneration due to the deficiency of microglial autophagy was associated with the regulation of NLRP3 inflammasome activation.

Cheng et al. (344) reported that loss of programmed death-1 (PD-1) exacerbates motor impairment in the MPTP model of PD by triggering microglial activation and neuroinflammation in mice. Utilizing a PD mouse model and PD-1 KO mice, these authors established distinct experimental groups: WT-CON, WT-MPTP, KO-CON, and KO-MPTP, which stand for wild-type-control, wild-type-1-methyl-4-phenyl-1,2,3,6-tetrahydropyridine, PD-1-KO-control, and PD-1-KO-MPTP, respectively. They examined motor dysfunction in animals, the dopaminergic neuronal injury, morphological distribution of PD-1-positive cells, glial activation, and the generation of inflammatory cytokines in the midbrains through motor behavior detection, immunohistochemistry, and western blot analyzes. In the WT-MPTP mouse model, a reduction in microglial cells positive for PD-1/Iba1 in the substantia nigra. was observed compared to WT-CON mice. Upon comparing the four experimental groups, PD-1 deficiency was associated with worsening motor dysfunction in animals, a decrease in tyrosine hydroxylase (TH) protein expression, and a reduction in TH-positive neuronal protrusions. Additionally, PD-1 deficiency amplified microglial activation, leading to increased production of proinflammatory cytokines such as iNOS, TNF-α, IL-1β, and IL-6. Moreover, PD-1 deficiency influenced the AKT and ERK1/2 expression and phosphorylation in the substantia nigra of the MPTP model.

Rocha et al. (345) demonstrated that in the rotenone model of PD, microglia-specific KO of NF-κB/IKK2 leads to an enhanced accumulation of misfolded α-synuclein. This effect is attributed to the inhibition of p62/sequestosome-1-dependent autophagy. The hypothesis was that inhibiting NF-κB signaling in microglia would alleviate overall inflammatory injury in lesioned mice. Analysis revealed a decrease in the microglia’s expression of sequestosome 1 (p62), an NF-κB-regulated autophagy gene. P62 is crucial for targeting ubiquitinated α-syn for lysosomal degradation. Despite an overall reduction in neurodegeneration, KO animals exhibited an increased accumulation of misfolded α-syn within microglia.

Metabolic reprogramming is now applied to elucidate the metabolic energy characteristics of microglia (346). Microglia exhibit distinct metabolic patterns (347). Activated microglia manifest unique metabolic profiles in glucose, amino acid, iron, and lipid metabolism. Furthermore, mitochondrial dysfunction may contribute to the metabolic reprogramming of microglia through the activation of various signaling mechanisms. The functional alterations in microglia resulting from metabolic reprogramming can induce changes in the brain microenvironment, which is pivotal in promoting neuroinflammation or facilitating tissue repair. The involvement of microglial metabolic reprogramming in the pathogenesis of PD has been substantiated. Effective reduction of neuroinflammation and preservation of dopaminergic neurons can be achieved by inhibiting specific metabolic pathways in M1 microglia or by transitioning M1 cells into the M2 phenotype (348).

Lu et al. (349) employed metabolic profiling to assess microglial energy metabolism, specifically oxidative phosphorylation and aerobic glycolysis. They utilized the mRFP-GFP-tagged LC3 reporter to investigate the role of transient receptor potential cation channel subfamily V member 1 (TRPV1) in microglial autophagy. In their study, they employed a mouse model of sporadic PD featuring α-synuclein preformed fibrils (PFF) in TRPV1^flox/flox^; Cx3cr1^Cre^ mice to examine the impact of TRPV1 activation on the neurodegenerative process. Their findings revealed that acute exposure to PFF induced microglial activation, leading to metabolic reprogramming from oxidative phosphorylation to aerobic glycolysis through the AKT–mTOR– hypoxia inducible factor 1α (HIF-1α) pathway. Over time, activated microglia transitioned into a state of chronic PFF tolerance, accompanied by widespread defects in energy metabolism. The authors demonstrated that boosting metabolism through treatment with the TRPV1 agonist capsaicin rescued metabolic impairments in PFF-tolerant microglia and addressed defects in mitophagy caused by the disruption of the AKT–mTOR–HIF-1α pathway. Furthermore, capsaicin was shown to attenuate phosphorylation of α-synuclein in primary neurons by enhancing phagocytosis in PFF-tolerant microglia *in vitro*. Finally, Lu et al. observed that behavioral deficits and the loss of dopaminergic neurons were accelerated in the PFF TRPV1^flox/flox^; Cx3cr1^Cre^ mouse model of sporadic PD. In general, this study contributes to a deeper comprehension of the complex mechanisms involved in microglial responses to α-syn pathology and provides valuable insights for the development of targeted therapeutic strategies for PD.

Iron emerges as a pivotal factor influencing microglial polarization in the context of PD. Activation of inflammasomes in microglia triggers a metabolic shift from oxidative phosphorylation to glycolysis, culminating in microglia polarization toward the proinflammatory M1 phenotype. This cascade ultimately leads to neuroinflammation and neurodegeneration. Furthermore, iron accumulation prompts microglia to adopt an inflammatory and glycolytic phenotype. Interestingly, the M2 phenotype microglia demonstrate increased susceptibility to ferroptosis, and inhibiting ferroptosis can potentially alleviate neuroinflammation (350). Ryan et al. (351) demonstrated that microglia derived from human-induced pluripotent stem cells cultivated in a tri-culture system exhibit heightened responsiveness to iron and vulnerability to ferroptosis—a form of cell death dependent on iron. Moreover, an excess of iron led to a notable shift in the transcriptional state of microglia, aligning with a transcriptomic signature identified in the postmortem brain microglia of individuals with PD. The data from these researchers also indicate that this microglial response plays a role in neurodegeneration, as the removal of microglia from the tri-culture system significantly postponed iron-induced neurotoxicity.

Gu et al. (352) observed a reduction in MT1 levels in microglia within the substantia nigra of the MPTP-induced PD mouse model. Activation of microglial MT1 significantly inhibited LPS-induced neuroinflammation, while the absence of microglial MT1 exacerbated inflammation. Metabolic reprogramming of microglia was identified as contributing to the anti-inflammatory effects of MT1 activation. Microglial MT1 activation reversed LPS-induced excessive aerobic glycolysis and OXPHOS. MT1 positively regulated the pyruvate dehydrogenase alpha 1 (PDHA1) expression, enhancing OXPHOS and suppressing aerobic glycolysis. Additionally, in LPS-treated microglia, MT1 activation reduced the toxicity of conditioned media to the dopaminergic DA cell line MES23.5.

**Figure 2:**
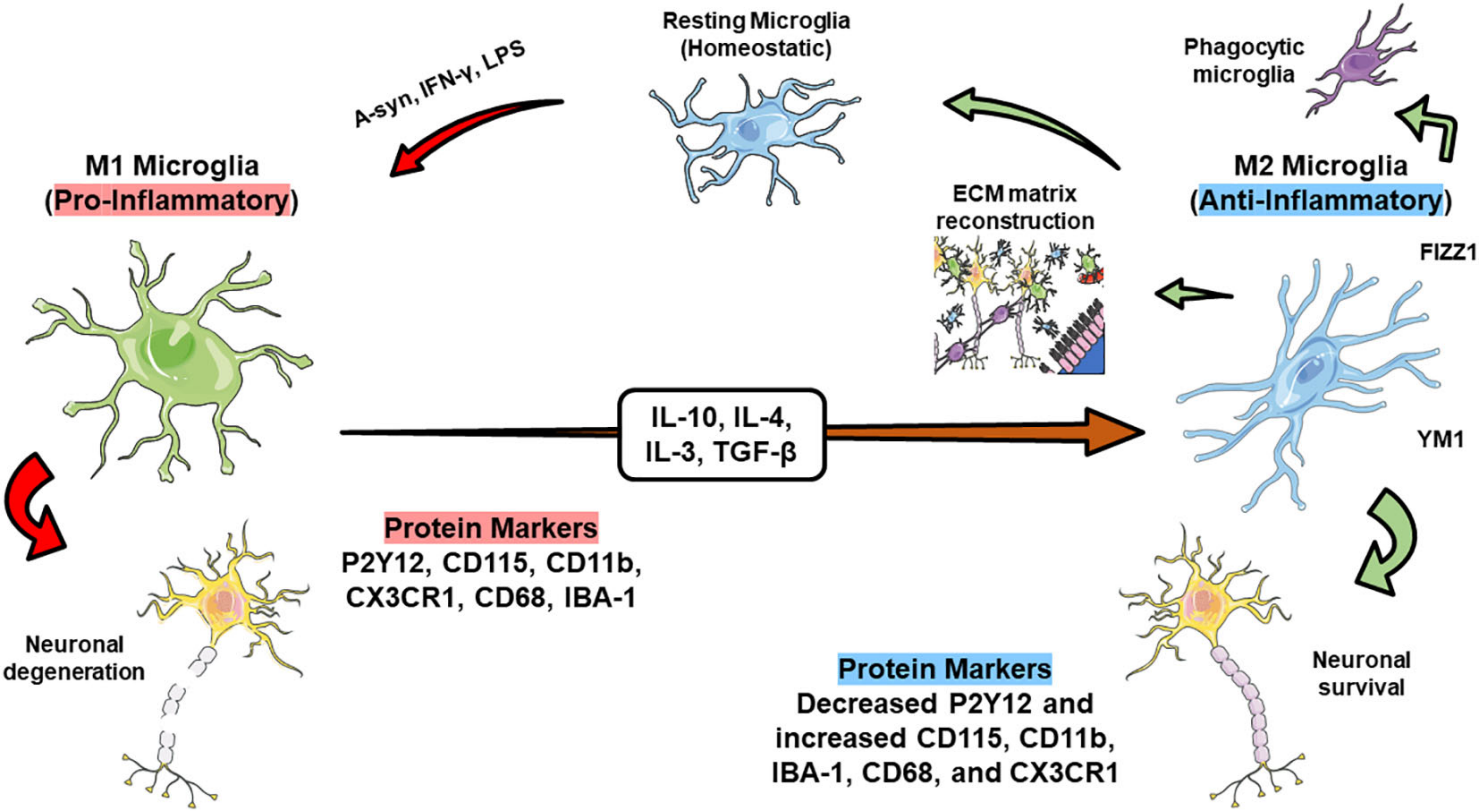
Microglia manifests distinct phenotypes, recognized as M1 and M2, in diverse circumstances. Typically, microglia exhibit a homeostatic phenotype that safeguards neuronal well-being through activities such as synaptic pruning, control of myelination, and the elimination of misfolded pathological proteins via neurogenesis and phagocytosis. Additionally, homeostatic microglia play a pivotal role in maintaining brain tissue equilibrium. Depending on environmental cues and encountered stimuli, microglia can transition into either the pro-inflammatory M1 phenotype or the anti-inflammatory M2 phenotype. In the presence of pathogenic molecules like LPS and IFN, or protein aggregates such as α-syn, microglia assume the pro-inflammatory M1 phenotype, releasing stressors like ROS and pro-inflammatory cytokines including IL-1β, iNOS, and TNF-α. Prolonged exposure to these inflammatory mediators may result in neuronal damage. Conversely, exposure to mediators like TGF-β, IL-4, IL-10, and IL-13 prompts microglia to shift from the M1 to the M2 phenotype. The M2 microglia phenotype contributes to processes such as phagocytosis, rebuilding the extracellular matrix (ECM), and promoting neuronal survival by secreting factors like YM1 and FIZZ1.

**Figure 2** depicts the varied phenotypes of microglia, namely M1 and M2, in response to different neurodegenerative conditions.

## 9 Contemporary approaches in clinical trials for managing neuroinflammation in neurodegenerative disorders

### 9.1 Current methods for targeting neuroinflammation in Alzheimer’s disease

**Table 1** outlines the key discoveries from the contemporary approaches in clinical trials to address neuroinflammation in AD.

**Table 1:** Principal findings from the modern strategies employed in clinical trials targeting neuroinflammation in Alzheimer’s disease.

Methods	Rationale	Clinical Trials
P38 MAPK inhibitor Neflamapimod	The activation of p38MAPKs by APP and Aβ in microglia and astrocytes results in the release of inflammatory cytokines. Simultaneously, in neurons, p38α MAPK amplifies pathogenic p-tau, leading to neuronal damage (353)	(354–356)
SIRT1 activator Resveratrol	The expression of SIRT1 exerts a notable neuroprotective impact (357)	(358)
Insulin sensitizer NE3107	NE3107 selectively inhibits inflammation-induced activation of ERK and NF-κB by binding to extracellular signal-regulated kinase	(359)
Intranasal insulin	Intranasal insulin administration protects against harmful inflammatory alterations in cerebrospinal fluid, ameliorates immune function, and improves cerebral vascular integrity	(360)
Plasma fraction GRF6019	Plasma fraction GRF6019 demonstrated various positive effects on brain aging in mice, such as enhanced cognition, neurogenesis, synaptic density, and reduced neuroinflammation	(361)
Lysergic acid diethylamide	Lysergic acid diethylamide exhibits notable anti-inflammatory properties mediated by signaling through the 5-HT2A receptor	(362)
HClO-scavenger Anserine	The progression of AD involves neuroinflammatory responses with the production of HClO	(363)
Microglial modulator CHF5074	CHF5074 has been demonstrated in prior studies to enhance synaptophysin levels, mitigate the buildup of native hyperphosphorylated tau, and reduce brain GSK-3β levels (364)	(365)
*Ginkgo biloba* EGb 761 Standardized Extract	Recognized for its neuroprotective effects, EGb 761 is widely employed worldwide in the symptomatic treatment of cognitive disorders, showcasing strong antioxidant and anti-inflammatory properties (366)	(367)

AD, Alzheimer’s Disease; APP, β-amyloid precursor protein; ERK, extracellular signal-regulated kinase; GSK, glycogen synthase kinase; MAPK, mitogen-activated protein kinase; NF-κB, nuclear factor kappa B; SIRT, sirtuin.

#### 9.1.1 P38 MAPK inhibitor Neflamapimod

Activation of p38MAPKs by APP and Aβ in microglia and astrocytes led to the release of inflammatory cytokines. Meanwhile, p38α MAPK heightened pathogenic p-tau in neurons, contributing to neuronal damage. Currently, two experimental medications are undergoing clinical trials that inhibit p38 MAPKs: Neflamapimod (NCT03402659) and MW150 (NCT05194163) (353). Yet, information on Neflamapimod is already publicly available.

Alam et al. (354) explored the correlation between plasma phosphorylated tau at residue 181 (ptau181) and the response to Neflamapimod treatment in patients with Dementia with Lewy Bodies (DLB). The study aimed to assess the impact of the p38α kinase inhibitor Neflamapimod, targeting the cholinergic degenerative process in DLB, on individuals with different baseline levels of pretreatment plasma ptau181. Eighty-five participants with mild-to-moderate DLB, receiving cholinesterase inhibitors, underwent analysis, with 45 participants below and 40 above the 2.2 pg/mL cutoff for plasma ptau181 at baseline. Over the 16-week treatment period, comparing placebo with Neflamapimod 40 mg three times daily, participants below the cutoff demonstrated greater improvements in all evaluated endpoints compared to those above the cutoff. Furthermore, individuals below the ptau181 cutoff at baseline exhibited significant enhancement over placebo in various measures, including an attention composite measure (+0.42, 95% CI 0.07-0.78, p = 0.023, d = 0.78), the Clinical Dementia Rating Scale Sum of Boxes (-0.60, 95% CI -1.04 to -0.06, p = 0.031, d = 0.70), the Timed Up and Go test (-3.1 seconds, 95% CI -4.7 to -1.6, p < 0.001, d = 0.74), and International Shopping List Test-Recognition (+1.4, 95% CI 0.2-2.5, p = 0.024, d = 1.00).

Jiang et al. (355) conducted a preclinical and randomized clinical assessment of the p38α kinase inhibitor Neflamapimod for basal forebrain cholinergic degeneration. Ninety-one participants with DLB, all undergoing background cholinesterase inhibitor therapy, were randomly assigned in a 1:1 ratio to receive Neflamapimod 40 mg or matching placebo capsules. The medication was administered orally twice daily for individuals weighing less than 80 kg and thrice daily for those weighing more than 80 kg. In the clinical study, Neflamapimod did not demonstrate an effect on the primary endpoint, a cognitive test battery. However, improvements were observed on two secondary endpoints—functional mobility and a dementia rating scale—which align with an anticipated impact on Basal Forebrain Cholinergic Neurons (BFCN) function. Neflamapimod treatment was well-tolerated, with no instances of study drug-associated treatment discontinuations.

Prins et al. (356) conducted a 24-week double-blind, placebo-controlled phase 2 clinical trial evaluating the p38 alpha kinase inhibitor Neflamapimod in individuals with mild AD. Named the REVERSE-SD (Synaptic Dysfunction) study, this multi-center trial took place from December 29, 2017, to June 17, 2019. A total of 464 participants were screened, and 161 were randomized to receive either 40 mg Neflamapimod (78 participants) or a matching placebo (83 participants) orally twice daily. The study did not reveal significant differences between the treatment groups concerning primary or secondary clinical endpoints. However, Neflamapimod treatment led to significantly reduced cerebrospinal fluid (CSF) levels compared to placebo, particularly in T-tau [difference (95% CI): -18.8 (-35.8, -1.8); P=0.031] and p-tau181 [-2.0 (-3.6, -0.5); P=0.012], with a suggestive trend for neurogranin [-21.0 (-43.6, 1.6); P=0.068]. In pre-specified pharmacokinetic-pharmacodynamic (PK-PD) analyzes, subjects in the highest quartile of trough plasma Neflamapimod levels exhibited positive trends, compared to placebo, in Hopkins Verbal Learning Test-Revised (HVLT-R) and Wechsler Memory Scale (WMS). The incidence of discontinuation due to adverse events and serious adverse events (all considered unrelated) was 3% each.

#### 9.1.2 SIRT1 activator resveratrol

The activation of sirtuins, particularly sirtuin 1 (SIRT1), could represent a crucial molecular pathway for treating AD neuroinflammation. The deacetylase activity of SIRT1 is controlled by the NAD+/NADH ratio, linking cellular energy balance to epigenetic transcriptional regulation. Resveratrol, a potent activator of SIRT1 and a pharmacological mimic of caloric restriction (CR), is a polyphenol naturally occurring in red grapes, peanuts, and various other plant species. Analogous to the effects of CR, the administration of resveratrol to transgenic mouse models of Alzheimer’s disease results in reduced behavioral deficits and diminished CNS Aβ deposition with aging (368). In a retrospective analysis, Moussa et al. (358) examined stored CSF and plasma samples from a subgroup of AD subjects with baseline CSF Aβ42 <600 ng/ml (N = 19 resveratrol-treated and N = 19 placebo-treated). Compared to the placebo-treated group, resveratrol significantly reduced CSF matrix metalloproteinase (MMP)-9 and increased macrophage-derived chemokine (MDC), IL-4, and fibroblast growth factor (FGF)-2 at 52 weeks. Resveratrol also led to increased plasma MMP10 and decreased IL-12P40, IL-12P70, and regulated on activation, normal T cell expressed and secreted (RANTES) compared to baseline. In this subset analysis, resveratrol treatment mitigated declines in mini-mental status examination (MMSE) scores, changes in activities of daily living (ADL) scores (ADCS-ADL), and CSF Aβ42 levels during the 52-week trial, while tau levels remained unchanged. These findings collectively suggest that resveratrol reduces CSF MMP9, modulates neuroinflammation, and triggers adaptive immunity.

#### 9.1.3 Insulin sensitizer NE3107

NE3107, an orally administered small molecule with blood-brain permeability, is an anti-inflammatory insulin sensitizer. This compound binds to extracellular signal-regulated kinase and has demonstrated its ability to specifically hinder inflammation-induced activation of extracellular signal-regulated kinase (ERK) and NF-κB, thereby inhibiting the production of inflammatory mediators such as TNF-α without disrupting their normal regulatory functions (359). In this Phase III clinical study, conducted at multiple centers and employing a randomized design, Reading et al. investigated the safety and effectiveness of a 30-week treatment with NE3107 compared to a placebo in elderly individuals diagnosed with mild-to-moderate AD. A total of 316 patients were randomly assigned in a 1:1 ratio. The co-primary endpoints assessed cognitive function (ADAS Cog12) and functional and behavioral traits (ADCS CGIC) with promising results. The comprehensive final findings are expected to be published shortly. The study is registered under the identifier NCT04669028 on ClinicalTrials.gov.

#### 9.1.4 Intranasal insulin

In pilot clinical trials, intranasal insulin (INI) has displayed potential as an AD treatment. A recent phase 2 trial involving participants with mild cognitive impairment (MCI) or AD, treated with INI via two delivery devices, demonstrated improved CSF biomarker profiles and slower symptom progression compared to the placebo group. In the benefiting cohort, changes in CSF markers related to inflammation, immune function, and vascular integrity were measured and evaluated concerning changes in cognition, brain volume, and CSF amyloid and tau concentrations. The insulin-treated group exhibited increased CSF interferon-γ (p = 0.032) and eotaxin (p = 0.049), along with reduced interleukin-6 (p = 0.048) over the 12-month trial. In contrast, the placebo group showed increased CSF macrophage-derived chemokine (p = 0.083), and the insulin-treated group exhibited trends for increased interleukin-2 (p = 0.093). Remarkably different patterns of associations between changes in CSF markers and alterations in cognition, brain volume, and amyloid and tau concentrations were observed between the insulin-treated and placebo groups (360).

#### 9.1.5 Plasma fraction GRF6019

The plasma fraction GRF6019 exhibits various positive effects on brain aging in mice, including improved cognition, neurogenesis, synaptic density, and reduced neuroinflammation. Hannestad et al. (361) conducted a phase II, double-blind, placebo-controlled study to assess the safety, tolerability, and preliminary efficacy of GRF6019 in patients with severe AD. Patients (GRF6019: n = 18, Placebo: n = 8) were randomized 2:1 and received 250 mL intravenous infusions daily over five days. The primary endpoints included adverse event rates, tolerability, and change from baseline in cognitive and functional assessments. All patients completed the study without any deaths or serious adverse events. GRF6019 (44.4%) and placebo (37.5%) negative event rates were similar. The most common treatment-related adverse events in the GRF6019 group were mild, transient changes in blood pressure. While this trial lacked statistical power to detect differences in cognitive and functional assessments between treated groups, GRF6019 exhibited excellent safety, feasibility, and tolerability. Subsequent trials designed to elucidate the potential practical benefits of GRF6019 and related plasma fractions in severe Alzheimer’s disease are justified.

#### 9.1.6 Lysergic acid diethylamide

Studies indicate that psychedelics, such as lysergic acid diethylamide (LSD), possess significant anti-inflammatory properties mediated through 5-HT2A receptor signaling. This supports their potential therapeutic use in addressing neuroinflammation associated with neurodegenerative diseases (362). In this double-blind, placebo-controlled, randomized phase 1 study, Family et al. assigned volunteers to one of four dose groups (5 μg, 10 μg, 20 μg LSD, and placebo) and administered the given dose on six occasions every four days. Forty-eight healthy older volunteers (mean age = 62.9 years) received placebo (n = 12), 5 μg (n = 12), 10 μg (n = 12), or 20 μg (n = 12) LSD. LSD plasma levels were undetectable for the 5 μg group, while peak blood plasma levels for the 10 μg and 20 μg groups occurred at 30 minutes. The study found that LSD was well-tolerated, with the frequency of adverse events no higher than that for the placebo. Assessments of cognition, balance, and proprioception revealed no impairment. The results suggest the safety and tolerability of orally administered 5 μg, 10 μg, and 20 μg LSD every fourth day over 21 days, supporting further clinical development of LSD for the treatment and prevention of AD.

#### 9.1.7 HClO-scavenger anserine

Specifically, research indicates that neuroinflammatory responses involving HClO production contribute to AD progression (363). In this study by Masuoka et al., anserine, an HClO scavenger, was investigated to protect cognitive function in individuals with MCI. Thirty-six MCI participants were assigned to receive either 500 mg of anserine per day or a placebo for 12 weeks. Cognitive function was assessed using the MMSE at baseline and post-ingestion. Analysis of MMSE change scores for 30 subjects completing follow-up tests revealed a significant difference between the active arm (1.9 ± 2.0; n = 15) and the placebo arm (0 ± 2.8; n = 15) (p = 0.036). The significance increased after adjusting for daily anserine intake (p = 0.0176). The results suggest that anserine may safeguard elderly individuals with MCI from cognitive decline by suppressing myeloperoxidase (MPO)-mediated neuroinflammatory responses.

#### 9.1.8 Microglial modulator CHF5074

Ross et al. (365) conducted a 12-week, double-blind, placebo-controlled, parallel groups, ascending dose study involving 96 patients with MCI to assess the safety, tolerability, pharmacokinetics, and pharmacodynamics of CHF5074, a novel microglial modulator. Participants were divided into three cohorts receiving ascending, titrated doses of CHF5074 (200, 400, or 600 mg/day) or placebo. Vital signs, cardiac safety, neuropsychological performance, and safety clinical laboratory parameters were monitored. Plasma samples were collected for drug concentration analysis, measuring soluble CD40 ligand (sCD40L) and TNF-α. Optionally, CSF samples were collected to assess drug levels, β-amyloid1-42 (Aβ42), tau, phospho-tau181, sCD40L, and TNF-α after the last dose. Ten patients did not complete the study, and at the end of treatment, CSF levels of sCD40L and TNF-α were inversely related to CHF5074 dose. Plasma sCD40L levels in the 600-mg/day group were significantly lower than in the placebo group (p=0.010). While no significant differences were found in neuropsychological tests, a positive dose-response trend in executive function was observed in APOE4 carriers. This study demonstrates that CHF5074 is well-tolerated in MCI patients with a 12-week titrated treatment up to 600 mg/day and has a dose-dependent impact on CNS biomarkers of neuroinflammation.

#### 9.1.9 *Ginkgo biloba* EGb 761 standardized extract

Morató et al. (367) investigated the effects of EGb 761, the standardized extract of *Ginkgo biloba*, in MCI patients. Blood markers of inflammation and oxidative stress were analyzed. The study involved a single-center, parallel-group design with a 12-month follow-up for participants with MCI (Global Deterioration Scale, GDS = 3), followed by an additional 12-month extension. During the initial 12 months, participants were randomized into two arms: the study group (n = 50) received one daily tablet of EGb 761 (240 mg orally), while the control group (n = 50) did not receive EGb 761 but underwent the same assessments. After the first 12 months, the EGb 761-treated group continued treatment, and the control group was offered EGb 761 (240 mg orally). The ongoing study, involving 100 MCI patients (60% women), had a mean age of 73.1 years, and the mean time between symptom onset and MCI diagnosis was 2.9 years. The mean MMSE score was 26.7. The most frequent comorbidities included depressive and anxiety disorders, as well as vascular risk factors. Results for the first year of treatment are anticipated by 2023. EGb 761 is known for its neuroprotective effects. It is globally used for the symptomatic treatment of cognitive disorders, displaying robust antioxidant and anti-inflammatory activity in experimental models and clinical observational studies (366).

### 9.2 Current methods for targeting neuroinflammation in Parkinson’s disease

**Table 2** summarizes the main findings derived from modern approaches in clinical trials aimed at addressing neuroinflammation in PD.

**Table 2:** Key outcomes from contemporary strategies implemented in clinical trials focusing on neuroinflammation in Parkinson’s disease.

Methods	Rationale	Clinical Trials
Repetitive transcranial magnetic stimulation	rTMS has demonstrated anti-inflammatory effects by reducing pro-inflammatory cytokines such as IL-1β and TNF-α, while simultaneously increasing anti-inflammatory cytokines like IL-10 and BDNF in both cortical and subcortical tissues (369)	(370)
Cholinesterase inhibitors Donezepil	The regulation of neuroinflammation in PD is governed by cholinergic neurotransmission	(371)
Myeloperoxidase inhibitor AZD3241	Microglia express myeloperoxidase, an enzyme responsible for generating ROS, which plays a role in initiating neuroinflammation in the disease (372)	(373)

BDNF, brain-derived neurotrophic factor; IL, interleukin; PD, Parkinson’s Disease; ROS, reactive oxygen species; rTMS, repetitive transcranial magnetic stimulation; TNF, tumor necrosis factor.

#### 9.2.1 Transcranial magnetic stimulation

Aftanas et al. (370) documented the therapeutic impact of repetitive transcranial magnetic stimulation (rTMS) on neuroinflammation and neuroplasticity in patients with PD in a placebo-controlled study. Specifically, they investigated the effects of rTMS on the motor cortex (bilaterally) and the left prefrontal cortex (dorsolaterally) concerning the spontaneous and mitogen-stimulated synthesis of pro- and anti-inflammatory cytokines by blood cells, as well as the level of BDNF in the blood serum of PD patients. The rTMS group exhibited significantly more positive clinical dynamics (evaluated by the unified Parkinson’s Disease rating scale) than the placebo group, accompanied by a noteworthy reduction in the spontaneous production of pro-inflammatory cytokines IFNγ and IL-17A. However, rTMS did not produce a significant effect on serum BDNF.

#### 9.2.2 Cholinesterase inhibitors Donezepil

Cholinergic neurotransmission plays a regulatory role in neuroinflammation in PD (374). Sawada et al. (371) conducted a delayed-start study investigating the impact of donepezil on cognitive decline in non-demented PD patients. The study encompassed a 96-week randomized, placebo-controlled, double-blind phase 1, followed by a 24-week donepezil extension phase 2. The primary endpoint was the change in the MMSE at week 120. Ninety-eight patients were randomly assigned to the early-start (donepezil-to-donepezil) and delayed-start (placebo-to-donepezil) groups. Baseline MMSE mean scores were 27.6 and 28.0, respectively. While the MMSE change at week 120 favored the early-start group, the difference was insignificant. However, in apolipoprotein ϵ4 carriers, the MMSE declined, unlike in non-carriers, and the interaction effect (intervention × ϵ4 genotype) was highly significant (P < 0.001). When analyzed with this interaction, the difference became significant (group difference 1.95 [0.33 to 3.57], P = 0.018). Additionally, the MMSE decline slope in phase 1 was significantly better in the early-start group compared to the delayed-start group (P = 0.048).

#### 9.2.3 Myeloperoxidase inhibitor AZD3241

Myeloperoxidase, an enzyme that generates ROS, is expressed by microglia. AZD3241, a novel compound, serves as a selective and irreversible inhibitor of myeloperoxidase. The proposed mechanism of action for AZD3241 involves reducing OS, thereby diminishing sustained neuroinflammation (373). Jucaite et al. conducted a phase 2 randomized placebo-controlled multicenter positron emission tomography study with the aim of investigating the impact of 8 weeks of treatment with AZD3241 on microglia in PD patients. Participants with PD were randomly assigned to receive either orally administered AZD3241 at 600 mg twice a day or placebo (in a 3:1 ratio) for the 8-week duration. The study assessed the binding of (11)C-PBR28 to the microglia marker 18 kDa translocator protein using positron emission tomography at baseline, 4 weeks, and 8 weeks. In the AZD3241 treatment group (n = 18), the total distribution volume of (11)C-PBR28 binding to the translocator protein was significantly reduced compared to baseline at both 4 and 8 weeks (P < 0.05). The reduction in distribution volume across nigrostriatal regions at 8 weeks ranged from 13-16%, with an effect size of 0.5-0.6. Conversely, there was no overall change in the total distribution volume in the placebo group (n = 6). AZD3241 was found to be safe and well-tolerated. The observed reduction in (11)C-PBR28 binding to the translocator protein in the brains of PD patients following AZD3241 treatment supports the hypothesis that inhibiting myeloperoxidase affects microglia.

### 9.3 Current methods for targeting neuroinflammation in multiple sclerosis

**Table 3** outlines the primary outcomes obtained from contemporary methodologies in clinical trials designed to tackle neuroinflammation in MS.

**Table 3:** Key results from recent methodologies employed in clinical trials dedicated to addressing neuroinflammation in multiple sclerosis.

Methods	Rationale	Clinical Trials
Caloric restriction diet	Nutrition plays a crucial role in regulating inflammation both systemically and within the CNS, exerting significant influences on metabolism, immunity, and the composition of the gut microbiome (375)	(376)
Chaperone and activator of toll-like receptor 2 small molecule B-Crystallin	HspB5, a small heat shock protein, exhibits therapeutic effects in neuroinflammation, including MS, by acting as a molecular chaperone and activating protective responses in microglia and macrophages through Toll-like receptor 2	(377)
Inhibiting T lymphocytes through costimulatory blockade using the fusion protein abatacept, which targets CTLA4-Ig.	The application of the CTLA4-Ig fusion protein abatacept for T lymphocyte costimulatory blockade shows potential as a promising and potentially effective treatment against neuroinflammation in RRMS	(378)
Coconut oil and epigallocatechin gallate for natural therapies	Given that MS pathology encompasses neuroinflammation, mitochondrial alterations, and cellular oxidation, the use of a nutritional anti-inflammatory and antioxidant intervention involving coconut oil and epigallocatechin gallate can be effective in counteracting the disease	(379)
Natalizumab	A humanized monoclonal antibody binds to α4β1-integrin, leading to a decrease in the migration of immune cells from the blood across the blood-brain barrier into the CNS (380)	(381)

CNS, central nervous system; CTLA4-Ig, cytotoxic T lymphocyte-associated antigen-4-immunoglobulin; MS, Multiple Sclerosis; RRMS, relapsing-remitting Multiple Sclerosis.

#### 9.3.1 Caloric restriction diet

Rahmani et al. (376) conducted a pilot study to assess the impact of a twelve weeks intermittent caloric restriction diet on mitigating neuroinflammation in midlife individuals with MS. The study randomly assigned ten participants with relapsing-remitting MS to either a 12-week intermittent calorie restriction (iCR) diet group (n = 5) or a control group (n = 5). Measurements of cortical thickness and volumes were performed using FreeSurfer, cortical perfusion was assessed through arterial spin labeling, and neuroinflammation was evaluated using diffusion basis spectrum imaging. After 12 weeks of iCR, the iCR group exhibited an increase in brain volume in the left superior and inferior parietal gyri (p: 0.050 and 0.049, respectively) and the banks of the superior temporal sulcus (p: 0.01). Moreover, cortical thickness improvements were observed in the bilateral medial orbitofrontal gyri (p: 0.04 and 0.05 in right and left, respectively), the left superior temporal gyrus (p: 0.03), and the frontal pole (p: 0.008), among other regions. Cerebral perfusion decreased in the bilateral fusiform gyri (p: 0.047 and 0.02 in right and left, respectively) and increased in the bilateral deep anterior white matter (p: 0.03 and 0.013 in right and left, respectively). Neuroinflammation, as demonstrated by hindered and restricted water fractions (HF and RF), decreased in the left optic tract (HF p: 0.02) and the right extreme capsule (RF p: 0.007 and HF p: 0.003). The preliminary findings indicate that iCR may have therapeutic benefits, including enhancements in cortical volume and thickness, as well as the attenuation of neuroinflammation in midlife adults with MS.

#### 9.3.2 Chaperone and activator of Toll-like receptor 2 small molecule B-crystallin

van Noort et al. (377) assessed the therapeutic impact of Alpha B-Crystallin in individuals with MS. Acting as a molecular chaperone and activating Toll-like receptor 2-mediated protective responses in microglia and macrophages, the small heat shock protein alpha B-crystallin (HspB5) has demonstrated therapeutic effects in various animal models of neuroinflammation, including those for MS. Despite its positive effects, it’s important to note that HspB5 can also stimulate human antigen-specific memory T cells to release IFN-γ, a cytokine with documented detrimental effects in the context of MS. In this 48-week randomized, placebo-controlled, double-blind phase IIa trial, three bimonthly intravenous injections of 7.5, 12.5, or 17.5 mg HspB5 were determined to be safe and well-tolerated in patients with relapsing-remitting MS (RRMS). While predefined clinical endpoints did not show significant differences between the relatively small groups of MS patients treated with HspB5 or placebo, repeated administration, particularly of the lower doses of HspB5, led to a progressive decline in MS lesion activity monitored by magnetic resonance imaging, a trend not observed in the placebo group. Exploratory linear regression analysis demonstrated that this decline was significant in the combined group receiving either of the two lower doses, resulting in a 76% reduction in both the number and total volumes of active magnetic resonance imaging lesions at 9 months into the study (377).

#### 9.3.3 Fusion protein abatacept

Khoury et al. (378) conducted a randomized trial assessing the efficacy of abatacept (CTLA4-Ig) in treating RRMS. The use of the cytotoxic T lymphocyte-associated antigen-4-immunoglobulin (CTLA4-Ig) fusion protein abatacept for costimulatory blockade of T lymphocytes holds promise as a potentially effective anti-neuroinflammatory treatment for RRMS. The study, known as ACCLAIM (A Cooperative Clinical Study of Abatacept in Multiple Sclerosis), was a phase II, randomized, double-blind, placebo-controlled, multi-center trial. Of the planned 123 participants with RRMS, 65 were randomized in a 2:1 ratio to receive monthly intravenous infusions of either abatacept or placebo for 24 weeks. At 28 weeks, participants switched to the opposite treatment, with the final dose administered at 52 weeks. The primary endpoint was the mean number of new gadolinium-enhancing (Gd+) lesions, as determined by magnetic resonance imaging scans performed every 4 weeks. Results revealed no statistically significant differences in the mean number of new Gd+ magnetic resonance imaging lesions between the abatacept and placebo groups. Similarly, there were no statistically significant differences observed in other magnetic resonance imaging and clinical parameters related to RRMS disease activity. Throughout the trial, abatacept was well-tolerated by participants.

#### 9.3.4 Natural therapies with coconut oil and epigallocatechin gallate

Considering that the pathology of MS involves neuroinflammation, mitochondrial alterations, and cellular oxidation, implementing a nutritional intervention with anti-inflammatory and antioxidant properties, such as coconut oil and epigallocatechin gallate (EGCG), holds promise for effectively combating the disease. Cuerda-Ballester et al. (379) conducted a pilot study involving 51 MS patients to examine the effects of treatment with coconut oil and EGCG on gait and balance. Participants were randomly assigned to either an intervention group or a control group. The intervention group received a daily dose of 800 mg of EGCG and 60 ml of coconut oil, while the control group received a placebo. This regimen continued for 4 months, during which participants followed a Mediterranean isocaloric diet. The study assessed initial and final measurements, including quantitative balance (Berg scale), perceived balance (ABC scale), gait speed (10MWT), gait resistance (2MWT), muscle strength (measured with a dynamometer), and serum levels of β-hydroxybutyrate (BHB). The intervention group exhibited a significant improvement in gait speed, quantitative balance, and muscle strength in the right quadriceps, with an observed enhancement in gait resistance in both groups. Positive correlations were found between balance and gait scales. In conclusion, the administration of EGCG and coconut oil appears to enhance gait speed and balance in MS patients, even though the latter may not be subjectively perceived by the individuals. Additionally, these variables seem interrelated and contribute to overall functionality. Although this study did not directly evaluate neuroinflammatory markers, BHB is notable for its observed neuroprotective efficacy in conditions such as stroke and neurodegenerative diseases, as well as for its anti-inflammatory effects (382, 383).

#### 9.3.5 Natalizumab

Natalizumab, a humanized monoclonal antibody, binds to α4β1-integrin, leading to a decrease in the migration of immune cells from the blood across the blood-brain barrier into the CNS (380). To examine the progression of brain atrophy and its correlation with inflammatory activity in individuals with RRMS undergoing natalizumab treatment, Magraner et al. (381) conducted an 18-month clinical trial. Eighteen RRMS patients were prospectively monitored after initiating natalizumab therapy. Monthly evaluations included assessments for relapse signs, adverse events, or disability progression. Magnetic resonance imaging scans were conducted before natalizumab initiation and every six months thereafter. Cross-sectional calculations of T2 lesion volume (T2LV) and baseline and 18-month measurements of normalized brain volume (NBV) were performed using Steronauta® and SIENAx software, respectively. The Longitudinal Percentage of Brain Volume Change (PBVC) was estimated with SIENA. Results showed that natalizumab reduced the annual relapse rate (ARR) by 67% and cumulative contrast-enhancing lesions (CEL) by 87.5%. The global PBVC from baseline to 18 months was -2.5%, primarily occurring in the first six months. T2 lesion volume decreased from 1000 mm3 to 960 mm3 (p=0.006), and NBV decreased from 1.55×10(5) mm3 to 1.42×10(5) mm3 (p=0.025). The number of relapses before treatment correlated with PBVC during the first semester (Pearson’s coefficient -0.520, p=0.003), while basal CEL or baseline T2LV did not correlate with the rate of brain atrophy. Nine patients exhibited clinical or radiological inflammatory activity throughout the follow-up, and their PBVC was significantly higher in the first semester (p=0.002).

## 10 Innovative immunotherapeutic strategies for alleviating neuroinflammatory progression: bridging the gap from laboratory to clinical

De Vlaminck et al. (384) reported resident and infiltrating macrophages’ dynamic adaptability and outcomes in the progression and resolution of neuroinflammation. Brain-resident microglia and Border-Associated Macrophages (BAMs) are self-renewing cell populations. This study investigated the fate of microglia, BAMs, and infiltrating macrophages in neuroinflammation and its resolution. Neuroinflammation in mice was induced while the host was actively infected with *Trypanosoma brucei* parasites. Within the infection, fate mapping and single-cell sequencing revealed an accumulation of microglia around the ventricles and an expansion of epiplexus cells. Depletion experiments through genetic targeting demonstrated that resident macrophages played a role in the initial defense against parasites and subsequently facilitated the infiltration of monocytes across the brain barriers. These monocyte-derived macrophages, recruited from outside, outnumbered the resident macrophages and showed more remarkable transcriptional plasticity, adopting profiles indicative of antimicrobial gene expression. However, the situation reversed as the disease progressed to its final stages. The recruited macrophages were swiftly cleared, and no engrafted monocyte-derived cells remained in the brain parenchyma of the animals.

Conversely, resident macrophages gradually returned to a state of homeostasis. The overall findings of this study illustrate a complex phenomenon that neuroscientists rely on resident microglia, which showed limited long-term transcriptional changes, whereas BAMs exhibited more pronounced alterations. Brain-resident and recruited macrophages demonstrate distinct responses and dynamics during infection and resolution phases (384).

Stated differently, research endeavors like the one mentioned here pave the way in a domain closely aligned with immunomodulation. This field of immunology holds promise for potentially averting the onset and impeding the advancement of neuroinflammatory processes, thereby countering the emergence or progression of numerous neurological conditions, including neurodegenerative disorders. Regulating the immune system, whether on a systemic level or specifically within the CNS, involves multiple targets closely associated with microglia and peripheral immune cells that infiltrate the CNS during neuroinflammatory challenges.

### 10.1 Unleashing the therapeutic potential of interleukin-driven immunomodulation

Choi et al. (385) examined the involvement of IL-27-producing B-1a cells in alleviating neuroinflammation and autoimmune conditions affecting the CNS. Regulatory B cells (Breg cells) that produce IL-10 or IL-35 (i35-Breg) play pivotal roles in modulating immune responses within the brain, especially involving neuroinflammation. During neuroinflammatory processes, i27-Bregs aggregate in the CNS and lymphoid tissues, safeguarding against autoimmune diseases targeting the respective organs. In this study, the authors corroborated the hypothesis that i27- regulatory B cells (i27-Breg) immunomodulation significantly improves neuroinflammation in C57BL/6J, B6.SJL-Ptprc^a^ Pepc^b^/BoyJ (CD45.1) and B6N.129P2-Il27ra^tm1Mak^/J (Il27rα^−/−^) mice with encephalomyelitis. This was achieved through the heightened expression of inhibitory receptors (Lag3, PD-1), the suppression of Th17/Th1 responses, and the promotion of inhibitory signals that transform conventional B cells into regulatory lymphocytes secreting IL-10 and/or IL-35 in the brain and spinal cord. Moreover, i27-Breg exhibited *in vivo* proliferation and sustained IL-27 secretion in the CNS and lymphoid tissues, offering a therapeutic advantage over the administration of biologics (IL-10, IL-35) swiftly metabolized *in vivo*.

Kang et al. (386) reported that exosome-mediated IL-35 production mitigates neuroinflammation in C57BL/6J treated mice. CD138+ plasma cells were activated with anti-IgM/anti-CD40 antibodies to produce i35-Breg exosomes (i35-Exosomes). Subsequently, each mouse received a treatment of approximately 2 × 10^^10^ exosomes (30 μg/mouse). Given their low toxicity and immunogenicity, it is crucial not to underestimate the potential of exosomes in alleviating neuroinflammatory processes. The exosomes demonstrated anti-neuroinflammatory and anti-uveitis effects by promoting the proliferation of Treg cells that secrete IL-10 and IL-35, while concurrently inhibiting Th17 responses. The exosomes derived from i35-Bregs also inhibited CD4+ T cell proliferation and attenuated INF-γ secretion.

Similarly, Yu et al. (387) explored the anti-neuroinflammatory properties of IL-35 produced by Bregs in countering neuroinflammation induced by an autoimmune CNS disease in C57BL/6J mice. The authors found the recruitment and binding of BATF, along with IFN regulatory factors (IRF)-4 and IRF-8 transcription factors, to the AP1-IRF-composite elements (AICEs) within the Gene ResultIL12A interleukin 12A (il12a), Gene ResultEBI3 Epstein-Barr virus induced 3 (ebi3), and/or Gene ResultIL10 interleukin 10 (il10) loci within the mechanism associated with the anti-neuroinflammatory potential of the treatment, which strongly implies the BATF-IRF-4-IRF-8 complex crucial role in orchestrating the transcriptional programs responsible for IL-10 and IL-35 regulation in B cells and their roles in mitigating neuroinflammation. Hence, the transfer of an individual’s Bregs shows potential as a practical approach for treating autoimmune and neurodegenerative disorders linked to inflammation.

### 10.2 Chemokine (CXC) receptors signaling and expression: double-edged swords in neuroinflammation regulation and intervention with immunomodulation

Chung & Liao (388) reported that CXCR3 signaling in glial cells mitigates experimental autoimmune encephalomyelitis by limiting the development of a pro-Th17 cytokine environment and decreasing the infiltration of Th17 cells into the CNS. EAE serves as a mouse model for studying MS. Throughout this study, CXCR3^(-/-)^ mice were employed, as well as wild-type (WT) mice for control experiments. The results indicated that CXCR3^(-/-)^ mice displayed more severe EAE and exhibited a significant increase in CNS-infiltrating Th17 cells compared to WT mice. Otherwise, the adoptive-transfer experiments demonstrated that recipient CXCR3^(-/-)^ mice receiving Th17 cells derived from splenocytes of myelin oligodendrocyte glycoprotein (MOG)-immunized CXCR3^(-/-)^ mice or MOG-immunized WT mice consistently developed more severe EAE and had a notable increase in CNS-infiltrating Th17 cells compared to WT recipient mice receiving Th17 cells from the same source. Additionally, during EAE, the CNS of MOG-immunized CXCR3^(-/-)^ mice exhibited an elevated count of activated glial cells, with CXCR3-deficient glial cells displaying heightened expression levels of cytokine genes essential for Th17 expansion and recruitment. These findings reveal a previously unacknowledged function of CXCR3 signaling within glial cells, suppressing the proliferation of Th17 cells in the context of EAE.

Furthermore, the authors’ results underscore that, beyond its established role in immune cell recruitment, CXCR3 within CNS glial cells exerts a pivotal role in mitigating the pro-Th17 cytokine/chemokine environment during EAE, consequently curtailing Th17 cell proliferation within the CNS and attenuating disease progression. The described effects can be attributed to the potential for anti-neuroinflammatory action of CXC receptors. The observed pathways indicated that ERK activation triggered by CXCR3 signaling dampened the activation of NF-κB, a pivotal transcription factor crucial for the induction of IL-23 and CCL20, both necessary for Th17 cell expansion and recruitment and experimental autoimmune encephalomyelitis neuroinflammatory model (388).

Mueller et al. (389) evaluated that suppression of hyaluronan (HA) synthesis protects against CNS autoimmunity and augments CXCL12 expression within the inflamed CNS, alleviating neuroinflammation in a mice model of EAE through the administration of 4-methylumbelliferone (4MU), a widely recognized inhibitor of HA synthesis. HA plays proinflammatory roles in the context of CNS autoimmunity. It accumulates in demyelinated MS lesions, promoting antigen presentation and intensifying T-cell activation and proliferation. Further, HA initiates signaling via Toll-like receptors 2 and 4, which trigger the expression of inflammatory genes. Finally, HA also aids in lymphocyte adherence to vessels and facilitates CNS infiltration through the vascular endothelium. It is well-established that the expression of CXCL12, an anti-inflammatory chemokine, is diminished in the cerebrospinal fluid cells of MS patients and the spinal cord tissue during EAE. In this study, HA inhibited the production of CXCL12, while 4MU elevated spinal CXCL12 levels in both naive animals and during neuroinflammation. Objectively, HA inhibition by 4MU promoted CXCL12 synthesis. The elevation of this molecule conferred protection in active EAE of C57Bl/6 mice, reducing spinal inflammatory infiltrates and the infiltration of Th1 cells while promoting the differentiation of regulatory T-cells. In adoptive transfer EAE, the authors highlighted that administering 4MU to donor mice notably reduced the encephalitogenicity of lymph node cells. In other words, HA aggravates CNS autoimmunity by amplifying encephalitogenic T-cell responses and suppressing the protective chemokine CXCL12 in CNS tissue. The inhibition of HA synthesis with 4MU protects against MS inflammatory burden and could serve as a significant therapeutic avenue for MS but also other neuroinflammatory conditions.

Song et al. (390) reported that exosomal MicroRNA-181c-3p derived from cortical neurons suppresses neuroinflammation by reducing CXCL1 expression in astrocytes of a rat model with ischemic brain injury. Rat astrocytes were subjected to exosomes isolated from cortical neurons to examine their impact on the expression of chemokine (C-X-C motif) ligand 1 (CXCL1) and the ensuing inflammatory response. The results indicated that exosomal miR-181c-3p originating from cortical neurons conferred protective effects against astrocyte neuroinflammation by reducing CXCL1 levels. Exosomes isolated from cortical neurons subjected to oxygen-glucose deprivation (OGD) reduced the levels of CXCL1 and inflammatory factors in astrocytes. Moreover, these exosomes delivered miR-181c-3p to attenuate CXCL1 expression in astrocytes. Verily, CXCL1 upregulation significantly triggered inflammatory responses within the CNS. Objectively, miR-181c-3p targeted CXCL1 as its gene of interest. The miR-181c-3p mimic and siRNA delivery against CXCL1 (si-CXCL1) was demonstrated to suppress astrocyte inflammation by reducing CXCL1 expression and alleviating neuroinflammation.

### 10.3 Chilling chronicles: cold exposure and immunological reprogramming

Spiljar et al. (391) examined immune shifts across diverse compartments during cold exposure and demonstrated that this energetically demanding stimulus significantly alleviates active neuroinflammation in EAE. At baseline and in various inflammatory mouse models, cold exposure reduced MHCII expression on monocytes, thereby dampening T cell activation and mitigating their pathogenicity via monocyte modulation. Conversely, the authors’ results also demonstrated that the genetic removal of monocytes or their depletion via antibodies, or the introduction of Th1- or Th17-polarized cells through adoptive transfer, nullifies the cold-induced effects on T cells or EAE, respectively. These discoveries establish a mechanical connection between ambient temperature and neuroinflammation. They propose a dynamic interplay between the metabolic adjustments triggered by cold exposure and autoimmunity. They highlight an energetic trade-off that confers benefits for immune-mediated diseases and neuroinflammation intervention through immunomodulation and immunological reprogramming. This competition between cold-induced metabolic adaptations and autoimmunity illuminates a crucial aspect of the complex relationship between environmental factors and immune responses in the context of neuroinflammatory disorders.

### 10.4 Fibrin-targeting immunomodulation as a beacon of relief for neuroinflammation

Ryu et al. (392) demonstrated that immunomodulation targeting fibrin emerges as a safeguard against both neuroinflammation and neurodegeneration. The CNS experiences the activation of innate immunity and the deposition of blood-derived fibrin in conditions such as autoimmune disorders and neurodegenerative diseases, encompassing MS and AD. To alleviate neuroinflammation by fibrin-targeting immunomodulation, researchers harnessed the effect of the monoclonal antibody 5B8, which homes in on the elusive fibrin epitope γ377-395. This approach allowed them to specifically quell inflammation and oxidative stress triggered by fibrin while leaving the clotting process untouched. The findings revealed that 5B8 effectively dampened fibrin-induced NADPH oxidase activation and the expression of proinflammatory genes, therefore diminishing neuroinflammation within the CNS of the treated animals.

Furthermore, in animal models of both MS and AD, 5B8 successfully infiltrated the CNS, securing a bond with parenchymal fibrin. Its therapeutic administration also led to a reduction in the activation of innate immunity and mitigated neurodegeneration. Conclusively, fibrin-targeting immunomodulation effectively curbed both autoimmunity- and amyloid-induced neurotoxicity. This approach holds promise for potential clinical benefits without broad suppression of innate immunity or disruption of coagulation processes across a spectrum of neurological disorders (392).

Adams et al. (393) stated that the gamma377-395 peptide derived from fibrin, hinders the activation of microglia and mitigates recurrent paralysis in autoimmune diseases of the CNS. Mice with a knock-in of fibrinogen-gamma(390-396A) or receiving intranasal administration of a fibrinogen-derived inhibitory peptide (gamma(377-395)) to hinder the fibrinogen-Mac-1 interaction pharmacologically were applied in the conduction of this research. The results successfully pinpointed fibrinogen as a newfound controller of microglia activation. Fibrinogen, located in perivascular deposits within MS lesions, triggers microglial cells via Mac-1, initiating the conversion of microglia into phagocytes by activating Akt and Rho pathways. This study demonstrated that explicitly targeting the interaction between fibrinogen and the microglia integrin receptor Mac-1 (alpha(M)beta(2), CD11b/CD18) effectively mitigates EAE in mice without deleting the coagulation function. The intervention not only reduced microglia activation, therefore alleviating neuroinflammation, but also mitigated recurring paralysis. Targeting the gamma(377-395) fibrinogen epitope also holds promise as a potential therapeutic approach for conditions like MS and other neuroinflammatory diseases linked to blood-brain barrier compromise and microglia activation as the intervention impacts the inflammatory rather than the coagulation properties of fibrinogen.

## 11 Conclusions and future research directions

Our review findings suggest that interventions centered around interleukin-driven immunomodulation, chemokine (CXC) receptors signaling and expression, cold exposure, and fibrin-targeting approaches hold promising potential for alleviating neuroinflammatory processes across a spectrum of models encompassing conditions like MS, EAE, PD, and AD. While the findings from the studies are promising, immunomodulatory approaches often face limitations due to Immune-Related Adverse Events (irAEs), which involve an unintended activation and inflammatory response against the host’s healthy tissues. Therefore, the conduction of randomized clinical trials in this matter is mandatory. The goal is to elicit a targeted immune response against neuroinflammation within a human host. Recognizing the research in neuroinflammation intervention with innovative immunomodulation strategies is imperative. These research initiatives have explored diverse facets of this field, such as modulating microglial activation through numerous animal models to advance our understanding of neuroinflammation and pinpoint potential therapeutic avenues for mitigating neuroinflammatory processes.

The ongoing research efforts are laying the groundwork for prospective avenues of exploration within the realm of immunomodulation, interventions targeting microglial activity, and the regulation of neuroinflammation. These endeavors contribute to our current understanding and chart a course for future investigations in these crucial areas of study. Firstly, engaging in exploring and identifying novel molecular targets integral to controlling microglial activation and neuroinflammation is vital. This endeavor may encompass the application of sophisticated genomic and proteomic analyzes to unveil hitherto undiscovered components within the immunological pathways linked to neuroinflammation. By employing cutting-edge techniques, we can unravel hidden facets of molecular regulation, paving the way for a deeper understanding of the mechanisms governing microglial activation and neuroinflammatory processes. Secondly, creating individualized immunomodulatory strategies customized to the unique neuroinflammatory profiles of each patient is essential. Employ precision medicine methodologies, including genetic and biomarker profiling, to tailor treatment approaches, enhancing efficacy and minimizing potential side effects. This approach ensures a targeted and personalized therapeutic intervention that considers the distinct characteristics of each patient’s neuroinflammatory condition. In the realm of personalized medicine, the outcomes of single-cell RNA-seq can be leveraged to guide extensive small molecule screenings, facilitating the identification of compounds that specifically target cell populations associated with neurodegenerative conditions. By pinpointing vulnerabilities and dysregulated pathways at the cellular level through single-cell RNA-seq, scientists can formulate combination therapies that address various aspects of neurodegenerative disease pathology. This strategy has the potential to enhance the effectiveness of treatments and tackle the intricacies inherent in these multifaceted disorders. Moreover, data from single-cell RNA-seq can serve as a roadmap for endeavors in cellular reprogramming, with the goal of transforming or replacing damaged cell populations with robust and functional cells. This innovative approach holds promise for regenerative therapies, aiming to restore lost neuronal function in the context of neurodegenerative conditions.

Thirdly, the intricate relationship between the CNS’s metabolic processes and immune responses must be examined deeper. It is crucial to profoundly explore the impact of changes in cellular metabolism on microglial activation and their role in the development of neuroinflammation. Identifying and targeting specific metabolic pathways may present groundbreaking approaches to modulate the progression of neuroinflammation, offering innovative strategies to intervene in the underlying mechanisms governing this complex interplay. In this particular context, one could delve into the potential impact of cold therapy on diminishing the metabolic processes essential for facilitating healing responses.

Fourthly, it is imperative to utilize and implement state-of-the-art imaging technologies for the real-time visualization and monitoring of microglial activation and neuroinflammation. Incorporate advanced neuroimaging modalities, such as positron emission tomography (PET) and functional magnetic resonance imaging (fMRI), to elevate our comprehension of the spatial and temporal dynamics involved in immunological responses within the brain in the context of neuroinflammation in neurodegenerative disorders is essential. Fively, when addressing neurodegenerative diseases, it’s advisable to thoroughly explore the potential benefits of combining immunomodulatory methods with established therapeutic strategies. Prioritize identifying treatment plans that effectively utilize immunological and traditional neuroprotective mechanisms to optimize clinical outcomes. One way to attain this is by incorporating natural neuroinflammation modulators, like bioactive natural compounds, in conjunction with other immunomodulatory methods. Sixthly, researchers should explore the application of nanotechnology for delivering immunomodulatory agents specifically to affected brain regions within the contexts of different neurodegenerative disorders. The focus should be on developing nanocarriers that can cross the blood-brain barrier, facilitating targeted and controlled release of immunomodulators. This innovative approach aims to mitigate neuroinflammation while minimizing off-target effects.

Seventhly, researchers should also prioritize the influence of immunomodulatory interventions on patient-reported outcomes and the overall quality of life. In addition to conventional clinical endpoints, it is recommended to integrate patient perspectives and subjective measures. This comprehensive approach will provide a thorough assessment of the holistic effects of immunomodulation on individuals dealing with neuroinflammatory conditions. Ultimately, exploring neural stem cell (NSC) transplantation as a potential therapeutic avenue unveils encouraging findings in its ability to counteract neurodegeneration through a multifaceted approach. Notably, this intervention showcases positive impacts by generating essential neurotrophic factors crucial for neuronal health and facilitating the replacement of damaged cells. Moreover, the process demonstrates a noteworthy enhancement in neuronal plasticity, fostering the adaptability and resilience of neural networks. Additionally, the observed reduction in neuroinflammation further contributes to the overall positive outcomes associated with NSC transplantation. Consequently, the collective evidence supports the assertion that NSC transplantation is not merely a potential therapeutic strategy but indeed emerges as a promising and effective intervention in addressing the challenges posed by neuroinflammation in neurodegenerative conditions.

## Author contributions

LF: Conceptualization, Data curation, Formal Analysis, Funding acquisition, Investigation, Methodology, Project administration, Resources, Software, Supervision, Validation, Visualization, Writing – original draft, Writing – review & editing. JAD: Data curation, Methodology, Conceptualization, Formal Analysis, Validation, Funding acquisition, Resources, Visualization, Writing – review & editing. ACA: Data curation, Methodology, Conceptualization, Formal Analysis, Validation, Funding acquisition, Resources, Visualization, Writing – review & editing. KT: Validation, Writing – review & editing. CM: Writing – review & editing. CR: Writing – review & editing. LS: Writing – review & editing. DD: Writing – review & editing. MD: Writing – review & editing. MV: Writing – review & editing. EdS: Writing – review & editing. RJ: Writing – review & editing. IJ: Writing – review & editing. SB: Conceptualization, Data curation, Formal Analysis, Funding acquisition, Investigation, Methodology, Project administration, Resources, Software, Supervision, Validation, Visualization, Writing – original draft, Writing – review & editing.
